# Successful Targeting of the Warburg Effect in Prostate Cancer by Glucose-Conjugated 1,4-Naphthoquinones

**DOI:** 10.3390/cancers11111690

**Published:** 2019-10-30

**Authors:** Sergey A. Dyshlovoy, Dmitry N. Pelageev, Jessica Hauschild, Ksenia L. Borisova, Moritz Kaune, Christoph Krisp, Simone Venz, Yurii E. Sabutskii, Ekaterina A. Khmelevskaya, Tobias Busenbender, Vladimir A. Denisenko, Natalia D. Pokhilo, Lyubov N. Atopkina, Markus Graefen, Hartmut Schlüter, Valentin A. Stonik, Carsten Bokemeyer, Victor Ph. Anufriev, Gunhild von Amsberg

**Affiliations:** 1 Department of Oncology, Hematology and Bone Marrow Transplantation with Section Pneumology, Hubertus Wald-Tumorzentrum, University Medical Center Hamburg-Eppendorf, 20251 Hamburg, Germany; j.hauschild@uke.de (J.H.); moritz.kaune@stud.uke.uni-hamburg.de (M.K.); tobias.busenbender@stud.uke.uni-hamburg.de (T.B.); c.bokemeyer@uke.de (C.B.); g.von-amsberg@uke.de (G.v.A.); 2 G.B. Elyakov Pacific Institute of Bioorganic Chemistry, Far-East Branch, Russian Academy of Sciences, 690022 Vladivostok, Russia; pelageev@mail.ru (D.N.P.); borisovaksenia@mail.ru (K.L.B.); alixar2006@gmail.com (Y.E.S.); khea-96@mail.ru (E.A.K.); vladenis@piboc.dvo.ru (V.A.D.); nat-pokhilo@piboc.dvo.ru (N.D.P.); atopkina@mail.ru (L.N.A.); stonik@piboc.dvo.ru (V.A.S.); anufriev@piboc.dvo.ru (V.P.A.); 3 School of Natural Sciences, Far Eastern Federal University, 690091 Vladivostok, Russia; 4 Martini-Klinik, Prostate Cancer Center, University Hospital Hamburg-Eppendorf, 20251 Hamburg, Germany; graefen@martini-klinik.de; 5 Institute of Clinical Chemistry and Laboratory Medicine, Mass Spectrometric Proteomics, University Medical Center Hamburg-Eppendorf, 20251 Hamburg, Germany; c.krisp@uke.de (C.K.); hschluet@uke.de (H.S.); 6 Department of Medical Biochemistry and Molecular Biology, University of Greifswald, 17489 Greifswald, Germany; simone.venz@uni-greifswald.de; 7 Interfacultary Institute of Genetics and Functional Genomics, Department of Functional Genomics, University of Greifswald, 17489 Greifswald, Germany

**Keywords:** 1,4-naphthoquinones, castration-resistant prostate cancer, Warburg effect, mitochondria, proteomics

## Abstract

Treatment of castration-resistant prostate cancer (CRPC) remains challenging due to the development of drug resistance. The Warburg effect describes the ability of cancer cells to consume larger amounts of glucose compared to normal tissues. We identified derivatives of natural 1,4-naphthoquinones to be active in CRPC and further synthetically modified them via glucose conjugation to increase selectivity by Warburg effect targeting. Mechanisms of action were examined by quantitative proteomics followed by bioinformatical analysis and target validation. Four synthesized molecules revealed the highest selectivity towards human CRPC cells, which correlated with higher GLUT-1 activity and expression. The compounds were able to induce pro-apoptotic signs and to inhibit the pro-survival processes and mechanisms of drug resistance (i.e., AR-signaling and autophagy). Proteome analysis suggested a disruption of the mitochondria/oxidative phosphorylation, which was validated by further functional analysis: thus, mitochondria depolarization, elevated levels of cytotoxic ROS, an increase of Bax/Bcl-2 ratio as well as release of mitochondrial AIF and cytochrome C to cytoplasm were observed. In conclusion, glucose-conjugated 1,4-naphthoquinones show potent activity and selectivity in human CRPC exerted via mitochondrial targeting. The compounds can overcome drug resistance against current standard therapies and suppress pro-survival mechanisms. This unique combination of properties makes them new promising candidates for the treatment of CRPC.

## 1. Introduction

Despite continuous progress in the treatment of metastatic castration-resistant prostate cancer resistance development remains a major challenge during the course of treatment with decreasing response rates and reduced progression free and overall survival with each additional treatment line [1]. Thus, cross resistance has been observed between androgen receptor targeting therapies with a > 50% PSA decline of only 4% when abiraterone is applied after enzalutamide [2]. Conventional chemotherapies can cause severe side effects due to a lack of sensitivity and specificity limiting the use especially in patients with comorbid conditions, a poor performance status and/or high chronological age [3]. Consequently, there is a high medical need for novel anticancer drugs characterized by innovative mechanisms of action and increased selectivity in prostate cancer. 

Tumor cells exhaust more energy for proliferation than normal cells by consuming excessive amounts of glucose via overexpressed sugar binding and transporting receptors (mainly GLUTs) [4]. Interestingly, even in aerobic conditions the cancer cells prefer glycolysis followed by lactate fermentation over more energetically efficient oxidative phosphorylation. This process which was called “aerobic glycolysis” leads to the generation of extra amounts of metabolites, which may be beneficial for the rapidly proliferating cancer cells [5]. This phenomenon known as the “Warburg effect” attracts attention as one of the promising strategies for specific tumor targeting. Thus, binding of small cytotoxic “warhead” molecules to sugar residues may increase selectivity due to the higher uptake by cancerous cells [4,6]. Glufosfamide was the first sugar-conjugated compound which has been synthesized [7], successfully passed several clinical trials [8] and is currently undergoing Phase III trials in metastatic pancreatic cancer. Remarkably, the glycosylated derivatives of several cytotoxic drugs, e.g., chlorambucil, busulfan, docetaxel and paclitaxel have been found to be less toxic to normal cells than their parent aglycons [9]. Due to the chemical reactivity of the hydroxyl group at C1 of glucose (glycoside hydroxyl) most of these derivatives were synthesized as glycosides (i.e., conjugated at C1 position) [9]. For such prodrug compounds it has been shown that after entering the cells via GLUT receptors (mainly GLUT-1) the glycoside bond is hydrolyzed. Thus, the glucose residue is eliminated and consequently metabolized either via anaerobic or aerobic pathway (the latest involves Krebs cycle which takes place in the mitochondria matrix). While the unconjugated active aglycon (drug) further exerts its cytotoxic action inside the cancer cell [4].

Despite the progress in synthetic chemistry and computational biology up to 50% of the drugs used nowadays in clinic (particular in oncology) have been created on the basis of natural products [10]. These molecules are frequently characterized by unique physiological properties. Synthetic modification and structure optimization may further increase selectivity and decrease toxic side effects [10]. In the current study, we identified derivatives of several natural compounds with a 1,4-naphthoquinone structural moiety which showed promising cytotoxic activity in CRPC cell lines harboring different resistance patterns. Remarkably, a quinone moiety is present in different standard chemotherapies, e.g., dactinomycin, mitomycin-C and anthracyclines [11]. 1,4-Naphthoquinones, being the most important and widely distributed chemical class in the quinones, exhibit anticancer as well as antiallergic, antibacterial, antifungal, anti-inflammatory, antithrombotic, antiplatelet, antiviral, lipoxygenase, radical scavenging and anti-ringworm activities [11]. Despite the pronounced anticancer activity the mechanism of action is still poorly understood [11,12]. Inhibition of topoisomerase-II [13,14] as well as formation of semiquinones and superoxide radicals resulting in dsDNA breaks [15] have been reported for some of these compounds (reviewed in [11,12]). Recently, we showed that cell death induced by 1,4-naphthoquinones may have a p53-independent character [16]. 

Thus, in order to increase selectivity of the identified 1,4-naphthoquinones by Warburg effect targeting we further generated glucose derivatives of these compounds. For this purpose we used a new modified alkylation reaction of 2-hydroxy-1,4-naphthoquinones with 6-deoxy-6- iodo-1,2:3,5-di-O-isopropylidene-α-D-glucofuranose. This reaction is a more efficient variant compared to transesterification of methoxynaphthazarin derivatives, which has been recently reported by us [16,17]. Thus, we were able to synthesize chimera molecules which possess a 1,4-naphthoquinone “warhead” moiety and a glucose residue, conjugated to each other via unusual non-glycoside bond. Three groups of the molecules were generated, namely diprotected [diisopropylidene derivatives which have no unsubstituted (no free) hydroxyl groups in the glucose moiety, e.g., compound 2], monoprotected [monoisopropylidene derivatives which have two unsubstituted hydroxyl groups in the glucose moiety, e.g., compound **3**) and unprotected conjugated [free-glucose derivatives, glucose moiety has four unsubstituted hydroxyl group, e.g., compound **4**]. Here, we report on the synthesis, anticancer activity and mechanism of action as well as on Warburg effect guided selectivity in CRPC of new quinone-carbohydrate conjugates *in vitro*.

## 2. Results

### 2.1. Activity of 2-Methoxy-1,4-Naphthoquinones in Human Drug-Resistant Prostate Cancer Cells

**Figure 1:**
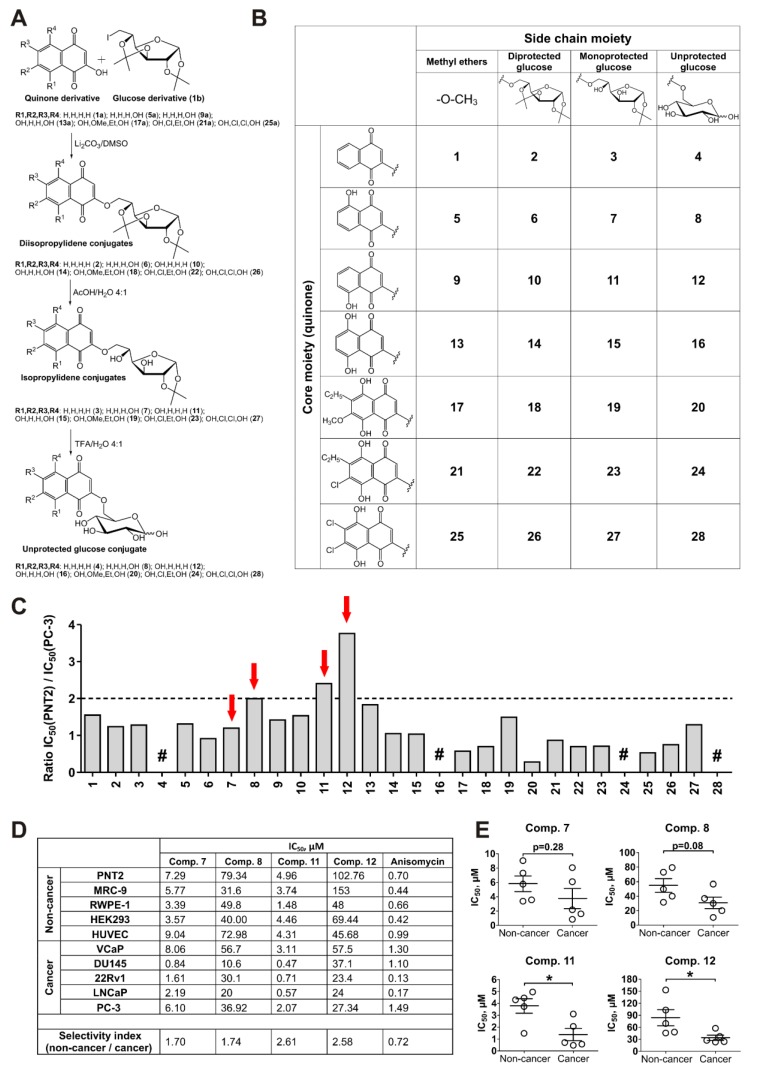
Cytotoxic activity of the synthesized compounds in cancer and non-cancer cells. (**A**,**B**) The scheme of the synthesis (**A**) and the structures (**B**) of the investigated conjugates. (**C**) Selectivity index values–the ratio of IC_50_ in PNT2 cells to IC_50_ in PC3 cells. “#”–IC_50_ values towards the tested cell lines were >100 µM. The IC_50_s values are correspondent to those presented in Table S4. (**D**,**E**) The cytotoxicity profile of the compounds **7**, **8**, **11**, and **12** in prostate cancer and non-cancer cells. IC_50_s were evaluated after 48 h of treatment using MTT test. Each dot represents IC_50_ towards certain human cell line (**E**). Anisomycin was used as a reference substance. Statistical significance: * *p* < 0.05 (Student’s *t*-test).

In continuation of the search for new natural compounds and their synthetic derivatives capable of overcoming the resistance of drug-resistant prostate cancer, we identified several 2-methoxy-1,4-naphthoquinones to be active in human prostate cancer PC-3 cells, known to be docetaxel-resistant and hormone-independent (due to the absence of AR expression) (compounds **1**, **5**, **9**, **13**, **17**, **21**, and **25**; Tables S2 and S4). However, the cytotoxicity of these compounds was comparable in malignant PC-3 cells and in human prostate non-cancer PNT2 cells (Table S4). Thus, we further modified these molecules and synthesized a library of 21 compounds containing a conjugated quinone and glucose moieties, linked via a non-glycoside bond (Figure 1A,B). These synthetic modifications have been postulated to increase the selectivity towards human cancer cells due to Warburg effect targeting.

### 2.2. Synthesis of the 6-O-(1,4-Naphthoquinone-2-yl)-D-Glucose Conjugates

For the synthesis, the small bioactive natural compounds lawsone (1a), the derivatives of juglone (**5a, 9a**) and naphthazarin (**13a, 17a, 21a** and **25a**) were chosen as the starting “warhead” molecules (Figure 1A, Table S1). A novel effective reaction of 2-hydroxy-1,4-naphthoquinones with 1,2:3,5-di-O-isopropylidene-6-deoxy-6-iodo-α-D-glucofuranose (**1b**) was applied to conjugate the quinone and D-glucose moieties. Monoprotected (monoisopropylidene) glucose conjugates (**3, 7, 11, 15, 19, 23, 27**) were prepared by acetic acid hydrolysis of diprotected (diisopropylidene) glucose conjugates (**2, 6, 10, 14, 18, 22, 26**). Conjugates, containing unprotected glucose (4, 8, 12, 16, 20, 24, 28), were synthesized by trifluoroacetic acid hydrolysis of monoprotected glucose conjugates. For further biological studies, we used the methyl ethers of the corresponding quinone derivatives as “reference” compounds–molecules containing active 1,4-naphthoquinone core and non-glucose substituted (compounds **1, 5, 9, 13, 17, 21, 25**).

### 2.3. Screening for Cytotoxicity and Selectivity to Human Prostate Cancer Cells

Next, we performed examinations of cytotoxicity and selectivity against human drug-resistant prostate cancer cells. We further examined the newly synthesized diprotected (**2, 6, 10, 14, 18, 22,** and **26**), monoprotected (**3, 7, 11, 15, 19, 23**, and **27**) and unprotected glucose conjugates (**4, 8, 12, 16, 20, 24**, and **28**), the corresponding methyl ethers as reference compounds (**1, 5, 9, 13, 17, 21**, and **25**) as well as anisomycin (positive control). Therefore, the same biological model was used–namely prostate cancer PC-3 cells and prostate non-cancer PNT2 cells.

In general, the selectivity index (SI) for glucose-quinone conjugates containing unprotected sugar residue (e.g., **8** and **12**) was higher than for the related methoxylated compounds without a sugar moiety (e.g., **5** and **9**) as well as mono- (e.g., **7** and **11**) or diprotected derivatives (e.g., **6** and **10**) (Figure 1C). However, their absolute cytotoxicity was significantly lower than of the other compounds belonging to the same structural group and IC_50_s often exceeded 100 µM (for details see Supplementary Information, Table S4). Nevertheless, we identified two derivatives, **8** and **12**, with unprotected glucose residues, which revealed SI ≥ 2 and IC_50_ < 100 µM. For further examinations we chose these substances as well as compounds **7** and **11**–monoprotected glucose conjugates residues belonging to the same structural groups like **8** and **12**. Compounds **7** and **11** showed high absolute cytotoxicity and rather high SI values (Figure 1B,C, Table S4).

The four selected compounds **7, 8, 11**, and **12** were then examined in a panel of human prostate cancer cell lines with varying levels of drug resistance as well as in five human non-cancer cell lines to gain further information on selectivity. Among others, PC-3 and DU145 (docetaxel-resistent, androgen-independent), 22Rv1 and VCaP (docetaxel-sensitive, androgen-independent, androgen receptor variant 7 (AR-V7) positive), and LNCaP (docetaxel-sensitive, androgen-dependent) prostate cancer cell lines were examined [18,19]. All four compounds exhibited selective cytotoxicity to prostate cancer cell lines. However, the activity and selectivity of 8-hydroxyquinone-2-yl (3-hydroxyjuglone) derivatives **11** and **12** was higher than of 5-hydroxyquinone-2-yl (2-hydroxyjuglone) derivatives **7** and **8** (Figure 1D,E). Of note, anisomycin used as a positive control was more cytotoxic in non-cancer cells and no selectivity was found (Figure 1D).

### 2.4. Cytotoxicity of the Synthesized Compounds Correlates with GLUT-1 Expression and Glucose Uptake Rate in Cancer Cells

**Figure 2:**
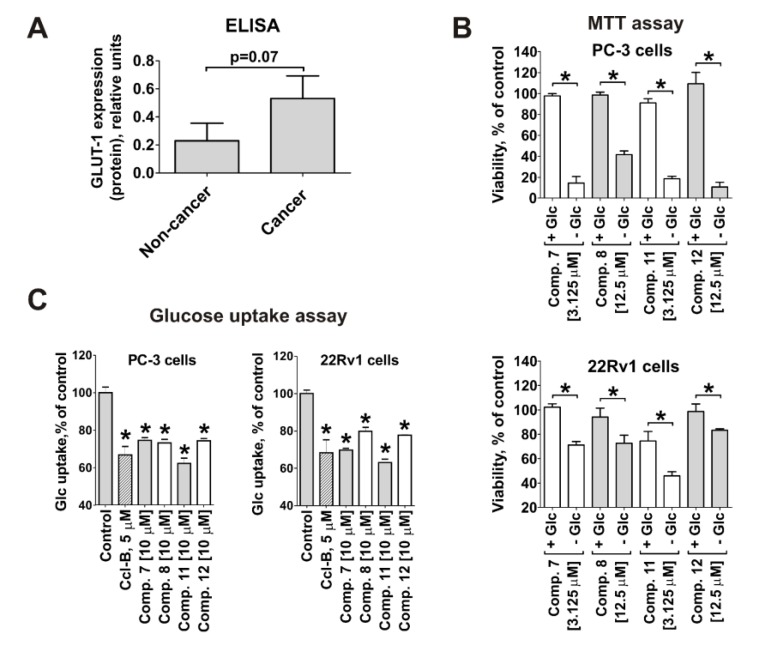
The correlation of cytotoxic activity of the synthesized compounds with cellular glucose uptake. (**A**) The expression of the glucose transporter 1 (GLUT-1) in five human prostate cancer cell lines versus five human non-cancer cell lines. The expression level was measured by ELISA. (**B**) Viability of PC-3 and 22Rv1 cells treated with the synthesized compounds at indicated concentrations in 10% FBS/RPMI media with or without 2 g/L glucose (Glc). The cytotoxic activity was measured by MTT test after 48 h of treatment and 12,000 cells/well. (**C**) Effect of the compounds on glucose uptake in PC-3 and 22Rv1 cells. Glucose uptake in viable (propidium iodide negative) cells was measured using 2-NBDG reagent and flow cytometry technique after 24 h of treatment. Cytochalasin B (Ccl-B, 5 µM) was used as positive control. In all the experiments cells were treated for 48 h. Statistical significance: * *p* < 0.05 (Student’s *t*-test, sections **A** and **B**; or ANOVA followed by a post-hoc Dunnett’s test, section **C**).

Next, we evaluated whether the anticancer activity and selectivity of the compounds correlate with glucose transporter-1 (GLUT-1) expression, as initially postulated. As expected, the mean GLUT-1 protein expression was 2.3-fold higher in prostate cancer cell lines than in non-cancer lines measured by ELISA (Figure 2A). The deletion of glucose from the culture media caused increased cytotoxicity of the tested compounds in both PC-3 and 22Rv1 cells (Figure 2B). Moreover, glucose uptake in human prostate cancer PC-3 and 22Rv1 cells was inhibited by the synthesized compounds (Figure 2C). These results could be well explained by the competitive binding of glucose and the investigated substances to GLUT-1. This is another strong evidence that the synthesized conjugates reveal affinity to GLUT-1 and are capable of targeting this pathway.

### 2.5. Effect on Prostate Cancer Cell Proliferation, Colony Formation and Viability

We have further examined the anticancer *in vitro* activity of the most promising compounds in human drug-resistant 22Rv1 cells. 22Rv1 cells express both AR full length (AR-FL) as well as AR-V7, whereas PC-3 cells are AR-FL- and AR-V7-negative [20]. Thus, 22Rv1 cells are suitable for monitoring of the both AR-FL- and AR-V7-mediated signaling [20]. Therefore, for the further experiments 22Rv1 cells were chosen as a main model, which is probably more clinically-relevant in comparison with PC-3 cells. 

**Figure 3:**
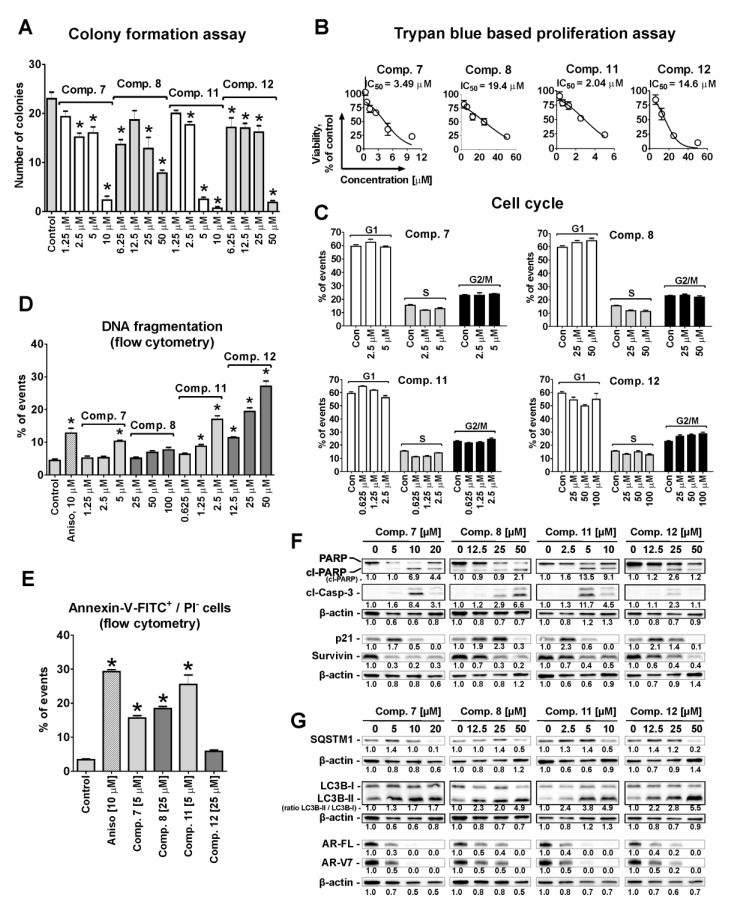
Cytostatic and proapoptotic activity of the selected conjugates. (**A**) Colony formation assay. The 22Rv1 cells were treated with the indicated concentrations of the compounds for 48 h, seeded in 6-well plates and incubated for 14 days. Cancer cell colonies were stained and counted by naked eye. (**B**), Cell viability and IC_50_s estimated in 22Rv1 cells using the trypan blue exclusion assay after 48 h of treatment. (**C**–**E**), Flow cytometry analysis of 22Rv1 cells treated with the investigated compounds for 48 h. (**C**,**D**), PI single staining: analysis of cell cycle. Apoptotic cells were detected as sub-G1 population (**D**). (**E**), Annexin-V-FITC/PI double staining. Cells appeared in low right quadrant (Annexin-V-FITC^+^/PI^-^) were considered to undergo early apoptosis. Flow cytometry data were analyzed and quantified using the Cell Quest Pro software. (**F**,**G**), The Western blotting analysis of the expression of pro- and anti-apoptotic proteins (**F**) as well as autophagy and AR-signaling related proteins (**G**) in 22Rv1 cells after 48 h of treatment. β-actin was used as a loading control. Cells treated with 10 µM of anisomycin (Aniso) for 48 h were used as a positive control. Statistical significance: * *p* < 0.05 (ANOVA followed by a post-hoc Dunnett’s test). Detailed information of Figure 3F,G (Western blotting) can be found at Figure S1.

Thus, all four selected conjugates **7, 8, 11**, and **12** were able to suppress cancer cell colony formation of AR-V7-positive 22Rv1 cells from single cells treated with non-cytotoxic concentrations for 48 h (Figure 3A). The anti-proliferative activity was determined by trypan blue exclusion assay (Figure 3B). Interestingly, the IC_50_s determined with trypan blue exclusion assay for compounds **7** and **11** (monoprotected glucose conjugates) (Figure 3B) were higher in comparison with the values estimated using MTT test, whereas for compounds **8** and **12** (unprotected glucose-quinone conjugates) the values were comparable. It is known that MTT tests access the metabolic activity of the cells [21], whereas trypan blue exclusion assays differentiate between the cells with intact or disrupted cellular membrane [22]. This suggests that monoprotected glucose conjugates **7** and **11** may primary suppress cancer cell metabolism, while non-protected glucose conjugates **8** and **12** may have higher membranotropic activity. Of note, no effect on the cell cycle progression was observed for 22Rv1 cells treated with any of the four tested compounds (Figure 3C).

The apoptosis-inducing activity of the compounds was examined by flow cytometry. Thus, a dose-dependent DNA fragmentation (Figure 3D) as well as phosphatidylserine externalization (Figure 3E) was detected in treated prostate cancer cells, indicating an apoptosis induction. In line with these results, the western blotting analysis of 22Rv1 cells treated with the different compounds for 48 h revealed dose-dependent induction of PARP and caspase-3 cleavage (apoptotic markers, Figure 3F), as well as down-regulation of the anti-apoptotic protein survivin (Figure 3F). Additionally, up-regulation of pro-apoptotic p21 was observed under the treatment followed by its down-regulation/degradation at higher cytotoxic concentrations (Figure 3F).

### 2.6. Effect on Resistance Mediating Autophagy and Androgen Receptor Splice Variant-7 Expression

Autophagy contributes to survival of cancer cells, e.g., by mediating resistance to standard therapies [23,24]. Proteins LC3 (isoforms I and II) and SQSTM1 (or p62) are major players in this process and therefore are commonly used markers for autophagy alteration [25]. Up-regulation of LC3B-II may indicate either inhibition or activation of autophagy, while simultaneous accumulation of LC3B-II and SQSTM1 generally indicates inhibition of autophagy [25]. In 22Rv1 cells accumulation of both, LC3B-II and SQSTM1, was detected at pre-cytotoxic and cytotoxic concentrations of compounds **7, 8, 11**, and **12** (Figure 3G) indicating an inhibition of autophagy.

Androgen receptor (AR) signaling mediates survival and progression of human prostate cancer [26]. The inhibition of this pathway by AR-targeting drugs, e.g., abiraterone and enzalutamide, leads to suppression of cell growth and ultimately to cell death. Resistance to these drugs is mediated by different mechanisms, such as AR-V7 expression [27]. AR-V7 lacks the C-terminal ligand binding domain and is a constantly activated transcriptional factor promoting cancer cell growth [26,27]. Compounds **7**, **8**, **11**, and **12** downregulated both, AR full length (AR-FL) and AR-V7 in 22Rv1 after 48 h treatment (Figure 3G). This effect may contribute to the drug-induced cell death and may lead to the reduction of the cancer cells drug-resistance.

### 2.7. Analysis of Changes in Proteome of Cancer Cells under Treatment With Synthesized Compounds

**Figure 4:**
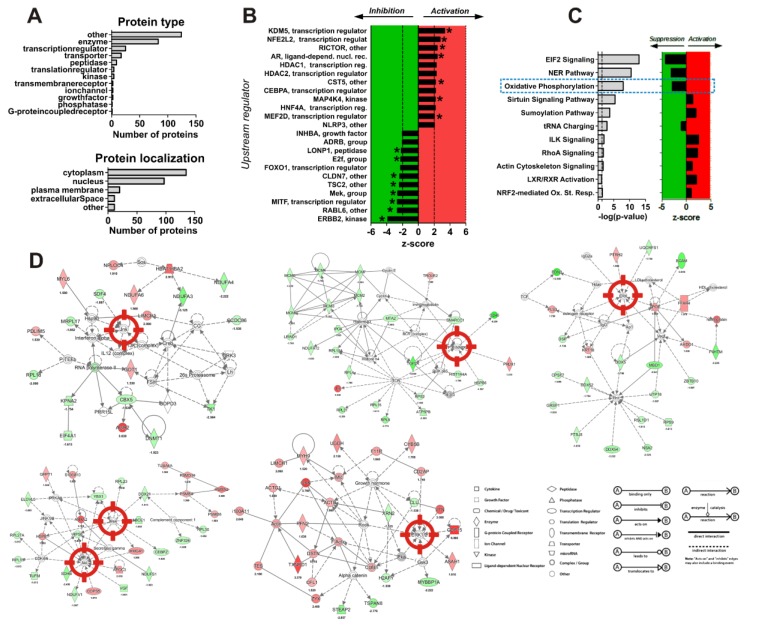

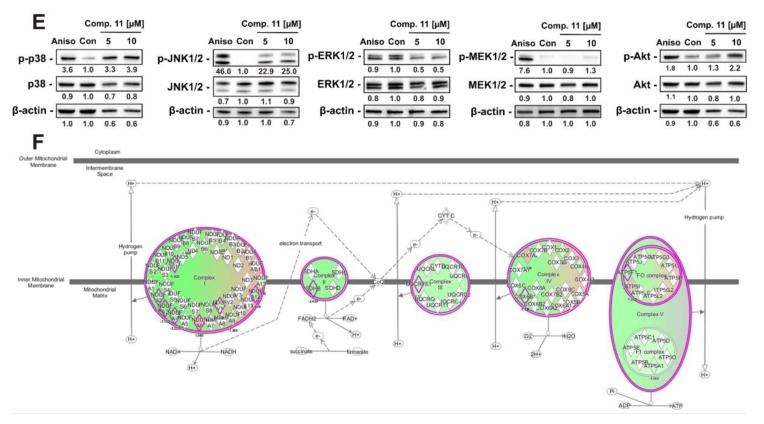
Proteomic and bioinformatical analysis of the effect of compounds 11 in human prostate cancer cells. 22Rv1 cells were treated with 5 µM of compound 11 for 48 h and the changes in proteome were analyzed with by LC-MS/MS. Bioinformatical analysis of the proteomics data was performed using Ingenuity Pathway Analysis (IPA) and *z*-score algorithm. Biological functions expected to be activated (*z*-score > 0, red area) or suppressed (*z*-score < 0, green area) are presented. (**A**) GO analysis. (**B**) Top predicted upstream target. *p*-value of overlap is indicated on the graph as * (*p* < 0.05). (**C**) Top predicted canonical pathways. (**D**) Hypothetical protein interaction networks constructed using IPA software. Relationships between proteins regulated upon exposure with compound 11, and proteins predicted to be involved in interactions, as well as relevant signaling pathways are presented. Red nodes represent up-regulated proteins, green nodes represent down-regulated proteins. Several kinases predicted by IPA software to be affected under the treatment are marked with red target sign. (**E**) Western blotting analysis of the kinases following 2 h treatment with compound 11. β-actin was used as a loading control. Detailed information of Figure 4E (Western blotting) can be found at Figure S2. (**F**) Representative schema of oxidative phosphorylation signaling built by IPA software. The molecule discovered by proteomics to be affected in cells under 22Rv1 treatment are indicated by color.

A global proteome screening approach was applied to further analyze the mode of action and molecular targets of the synthesized compounds. For this analysis the effect of the cytotoxic concentrations of compound **11** (most active conjugate in previous experiments) on the proteome of 22Rv1 cells was examined using the LC-MS/MS technique in data independent acquisition (DIA) mode. In total, 2163 proteins were quantified across all samples. 274 proteins had 1.5 ≤ fold change ≤ 1/1.5 and were significantly regulated (p ≤ 0.05) (see Supplementary Information, Table S5). We identified 92 proteins out of this pool with increased expressional levels (upregulation) and 182 with decreased expressional levels (downregulation) following the treatment. To analyze the proteomics data we used the Ingenuity Pathways Analysis tool (IPA, QIAGEN Bioinformatics, Redwood, CA, USA). Gene ontology, functional analyses as well as pathway network analyses were performed (Figure 4A–D). z-Score algorithm was used to predict the direction of alteration for the current activity or function: positive values indicate activation, negative values indicate suppression (Figure 4B,C). Interestingly, the majority of the proteins regulated under the treatment were enzymes localized in cytoplasm and nucleus (Figure 4A). The activity of several important cancer-relevant upstream regulator molecules was predicted to be affected (Figure 4B). Among them, up-stream tyrosine kinase receptor ErbB-2 [28], Rab-like protein 6 (RABL6) [29], microphthalmia-associated transcription factor (MITF) [30], which are involved in progression and development of several types of cancer were predicted to be suppressed (Figure 4B). In addition, canonical EIF2 and NER pathway as well as oxidative phosphorylation were predicted to be suppressed, while LXR/RXR, sirtuin, sumolaytion, ILK, RhoA, actic cytoskeleton signaling and NRF2-mediated oxidative stress response were activated (Figure 4C). Note, the IPA analysis predicted an activation of androgen receptor under the drug treatment (Figure 3G). However, we previously demonstrated the down-regulation of AR-FL as well as AR-V7 (Figure 3G). AR may be activated by phosphorylation, whereas the down-regulation/degradation of the total AR may eventually lead to the ultimate and general inhibition of AR signaling [31]. These events may appear independently from each other and thus will be further analyzed.

Five top hypothetical protein interaction networks were constructed using IPA software (Figure 4D). Treatment with compound **11** was predicted to affect kinases JNK1/2, p38, ERK1/2, MEK1/2, and Akt. Consequently, these kinases were placed in central positions of the networks (Figure 4D, marked with red target sign). To verify this hypothesis western blotting analyses were performed. As many stress kinases are known to be often activated shortly after stimulation, we used a short-term treatment of 2 h in order to validate the predicted alterations and elucidate their possible role as an early biochemical event following the treatment. Indeed, in line with the IPA analysis a pronounced activation of two main stress MAPKs–p38 and JNK1/2, as well as slight Akt activation were detected (Figure 4E), while pro-survival ERK1/2 was suppressed (Figure 4E). No significant alteration of MEK1/2 (an upstream kinase of ERK1/2) was observed (Figure 4E).

Interestingly, an oxidative phosphorylation (along with eIF2 signaling and NER pathway) was predicted to be significantly suppressed under the treatment (*z*-score = −2.887, *p*-value = 1.02 × 10^−8^; Figure 4F). Proteins which are important components of all five main complexes, Complex I (NADH-coenzyme Q oxidoreductase, genes *NDUFA4*, *NDUFV1*, *NDUFS1*, *NDUFA6*, *NDUFA12*, *NDUFA3*, *NDUFA2*), Complex II (succinate-Q oxidoreductase, gene *SDHB*), Complex III (Q-cytochrome c oxidoreductase, gene *UQCRFS1*), Complex IV (cytochrome c oxidase, genes *COX6B1*, *COX17*), and Complex V (ATP synthase, gene *ATP5PB*) were down-regulated by compound **11** (Figure 4F, Table S5). Thus, proteomics data strongly suggested the disruption of oxidative phosphorylation in cancer cells.

### 2.8. Mitochondria Are a Primary Target of Quinone-Glucose Conjugates 7 and 11 in Prostate Cancer Cells

**Figure 5:**
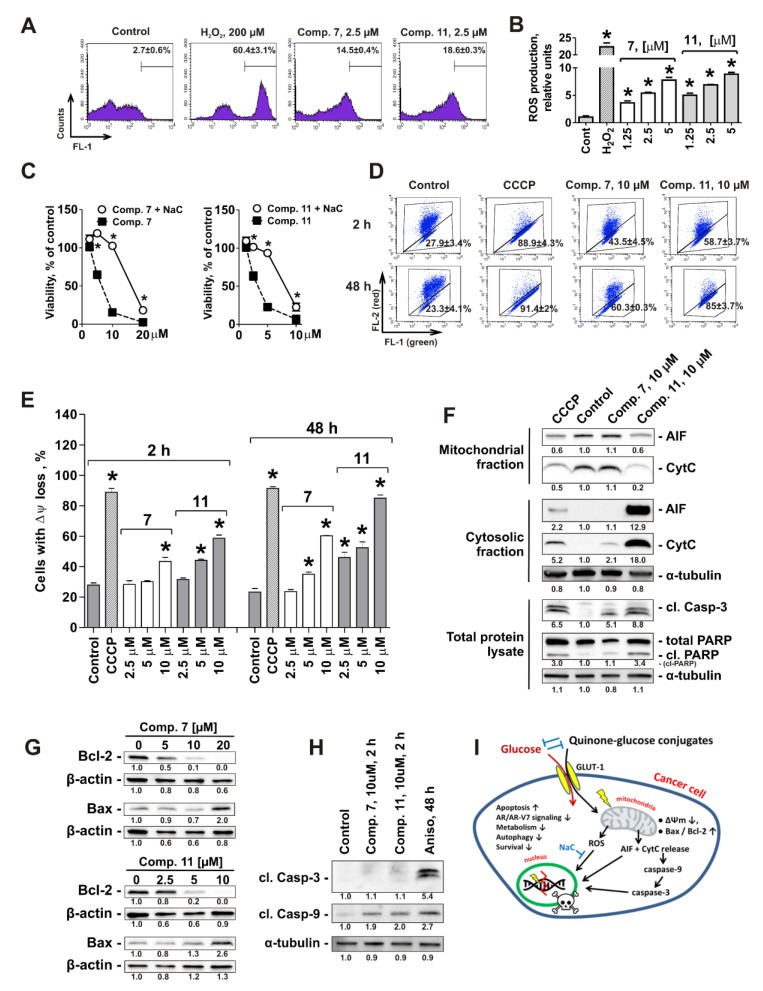
Quinone-glucose conjugates 7 and 11 primary target mitochondria of human prostate cancer cells. (**A**,**B**) Effect on ROS production in 22Rv1 cells. Cells were pretreated with CM-H_2_DCFDA followed by treatment with indicated concentrations of the investigated drugs for 2 h. Cells were harvested, analyzed by flow cytometry technique (**A**) and quantified using the Cell Quest Pro software (**B**). H_2_O_2_-treated cells were used as a positive control. (**C**) Effect of N-acetyl-L-cysteine (NaC) on the cytotoxicity of compounds 7 and 11. 22Rv1 cells were pre-treated with 1 mM NaC for 1 h and then co-treated with the investigated drugs for 48 h in FBS- and glucose-free RPMI media. Cell viability was measured by MTT test. (**D**,**E**) The induction of mitochondrial membrane potential (MMP, ΔΨm) loss by the drugs. 22Rv1 cells were treated for either 2 h or 48 h in PBS, harvested, stained with JC-1 and analyzed by flow cytometry technique. Numbers indicate the percentage of cells showing a drop in red fluorescence intensity, which corresponds to mitochondria depolarization. Cells showing ΔΨm loss have been quantified using Cell Quest Pro software (**E**). Cells treated with 50 µM CCCP for indicated time were used as a positive control. (**F**) 22Rv1 cells were treated with indicated concentrations of compounds **7** and **11** for 48 h. Proteins were isolated and fractionated using Cell Fractionation Kit (abcam), and the fractions were analyzed by Western blotting. Cells treated with 50 µM CCCP were used as a positive control. (**G**) Analysis of Bax and Bcl-2 expression in 22Rv1 cells following 48 h treatment. (**H**) Analysis of caspase-3 and -9 activation in 22Rv1 cells following 2 h and 48 h treatment. (**I**) Suggested mode of action of compounds **7** and **11** and other similar compounds. Statistical significance: * *p* < 0.05 (Student’s *t*-test, section **C**; or ANOVA followed by a post-hoc Dunnett’s test, sections **B** and **E**). Detailed information of Figure 5F–H (Western blotting) can be found at Figures S3–S5.

Oxidative phosphorylation, which was predicted by the global proteomic screening followed by the bioinformatical analysis to be suppressed under the drug treatment, takes place in the inner mitochondrial membrane [32]. Thus, we investigated the effect of compound **11**, as well as of structurally-related compound **7**, on mitochondria of human prostate cancer cells. First, the effect on the ROS level, which is often associated with the disruption of mitochondrial integrity (reviewed in [33]), was examined. Indeed, a remarkable and significant increase of the ROS level was observed already after 2 h of treatment in 22Rv1 cells (Figure 5A,B). Next, the impact of ROS alteration for cytotoxic activity of the investigated compounds was determined. Thus, cancer cells were treated with the drugs following pre-treatment with well-established antioxidant N-acetyl-L-cysteine (NaC, Figure 5C). Remarkably, pre-treatment with NaC significantly suppressed the cytotoxic activity of both compounds **7** and **11** (Figure 5C).

As the protein complexes which are involved in oxidative phosphorylation and potentially affected by the drugs are localized in the inner mitochondrial membrane (Figure 4F), we further checked the effects of conjugates **7** and **11** on ΔΨm using the fluorescence JC-1 dye and a flow cytometry technique. Indeed, a quick ΔΨm loss was detected in 22Rv1 cells after 2 h of treatment (Figure 5D,E). In line with this finding, we observed a release of mitochondrial proteins, namely apoptosis-inducing factor (AIF) and cytochrome C into the cytoplasm (Figure 5F). This was associated with an induction of two apoptotic markers, i.e., PARP- and caspase-3-cleavage (Figure 5F) and therefore may also contribute to induction of the drug-induced apoptosis. Remarkably, CCCP–a well-established mitochondrial oxidative phosphorylation uncoupler–exhibited comparable effects on ΔΨm and mitochondrial proteins release like compounds **7** and **11,** suggesting a similar mode of action (Figure 5D–F). Additionally, it has been shown that conjugates **7** and **11** induce up-regulation of Bax and down-regulation of Bcl-2, which may also contribute to the disruption of mitochondrial membrane permeability and ΔΨm loss (Figure 5G). Finally, it is important to note that the cleavage of caspase-9 in the cells treated with **7** and **11** was observed already after 2 h following the treatment and prior to the caspase-3 cleavage (which was detected only 48 h following the treatment) (Figure 5H). These data strongly suggest mitochondria as a primary target of the monoprotected glucose conjugates **7** and **11** (Figure 5I).

## 3. Discussion

Increased selectivity and efficacy can be achieved by linking cytotoxic anticancer drugs to specific target/carrier molecules [9]. Successful examples of this treatment strategy are antibody-drug conjugates, which target cancer cells due to increased expression of specific proteins [34], and folate conjugates, which utilize a commonly high expression of the folate receptor (FR) on the surface of different human cancer cells [35].

Warburg effect targeting is another promising strategy for the design and synthesis of new cytotoxic anticancer drugs. Here, the conjugation of sugar residues to cytotoxic drugs shall increase selectivity to cancerous tissues and reduce side effects in patients due to a higher sugar uptake by the cancer cells [4,9]. To date one sugar-conjugated molecule glufosfamide is in clinical development for the treatment of lung, ovary, pancreatic, brain and CNS cancer as well as soft tissue sarcoma [36]. In addition, different sugar-conjugated molecules are currently evaluated preclinically [4,9]. In prostate cancer Warburg effect was observed specifically in metastatic tumors which makes glucose-derived chemotherapeutics promising in the treatment of metastatic CRPC [37]. In our research, we found the derivatives of natural 1,4-naphtoquinone compounds to be active in drug-resistant prostate cancer cells. Preliminary data, however, have shown that these substances are also rather cytotoxic for non-cancerous cells, suggesting undesired side effects *in vivo*. Therefore, we utilized the Warburg effect in order to increase the selectivity of the compounds towards tumor cells. We synthesized hybrid molecules, which contain selected natural 1,4-naphtoquinone moieties conjugated with glucose residues via non-glycoside bond at C6 position. The non-glycoside nature of the conjugates was chosen in order to provide the highest affinity to glucose transporters located in the cellular membrane. It is known that the presence of free unsubstituted glycoside hydroxyl (at C1 position) is important for the stabilization of hydrogen bonding interactions with GLUT-1 and therefore is required for the successful cellular uptake of the sugar-conjugated molecules [38]. In contrast, the hydroxyl at C6 position of glucose molecule is not relevant for the hydrogen bond interactions and therefore it’s substitution may be more tolerable [38]. In fact, the affinity of glucose with substituted C6-OH to GLUT-1 was reported to be almost equal to its unsubstituted analogues [39]. Therefore, instead of a conventional glycosylation reaction (results in C1-substituted glucose) we applied a new reaction of alkylation of 2-hydroxy-1,4-naphthoquinones with 6-deoxy-6-iodo-1,2:3,5-di-O- isopropylidene-α-D-glucofuranose (results in a C6-substituted glucose). This reaction was more efficient in comparison with transesterification reaction of 2-methoxy-1,4-naphthoquinones, which was recently discovered by us [16,17,40]. Thus, we synthesized a series of 6-(1,4-naphtoquinonyl)-D-glucose conjugates containing diprotected, monoprotected, or unprotected glucose residues.

In line with our hypotheses the glucose-conjugated compounds revealed higher selectivity towards five human prostate cancer cell lines compared with five non-cancerous lines. The successful targeting of the Warburg effect by the newly synthesized glucose derivatives is strongly suggested by the following findings: (a) correlation of cytotoxicity and selectivity of the synthesized conjugates with higher expression of GLUT-1 in human prostate cancer cells; (b) inhibition of cytotoxicity of the conjugates by addition of glucose to the media; c) ability of the compounds to inhibit a glucose uptake by cancer cells. In general, the binding of the quinone core with the glucose moiety lead to an increased water solubility and selectivity. However, at the same time it resulted in an absolute cytotoxicity decrease when compared to methoxy derivatives, most probably due to the increased polarity of the molecule. It should also be noted that normal prostate cells are known to have an active glycolysis and also consume significant amount of glucose (reviewed in [37]). Thus, these cells may also be affected by the Warburg effect targeting therapeutics in vivo. However, frequently patients with advanced prostate cancer have had local treatment for their primary tumors leaving no vital tissue behind. In the patients cytoreduction of the remaining prostate tissue maybe a pleasant side effect in order to avoid local complications.

The four most promising compounds were selected for further examinations of activity and the mechanism of action. Namely, conjugates **11** and **12** belonging to the structural family of 8-hydroxy-1,4-naphtoquinone-2-yl (3-hydroxyjuglone) derivatives and containing monoprotected and free glucose, respectively, as well as conjugates **7** and **8**, which belong to the structural family of 5-hydroxy-1,4-naphtoquinone-2-yl (2-hydroxyjuglone) derivatives. These compounds exhibited pronounced anticancer activity, executed via induction of p21- and caspase-related apoptotic tumor cell death, and were able to suppress the colony formation of cancer cells (*in vitro* prototype of the anti-metastatic activity). Note, despite of the observed up-regulation of p21 no significant cell cycle arrest was found, which could be a cell specific phenomenon; whereas the p21 down-regulation detected at the higher concentrations of the drugs maybe a result of protein degradation driven by cell death associated processes. Interestingly, monoprotected conjugates **7** and **11** may have slightly different effects on cancer cells primarily targeting the metabolism, whereas free glucose containing conjugates **8** and **12** rather have a membranolitic activity. All four compounds effectively suppressed pro-survival processes, associated with drug resistance of prostate cancer cells. Thus, inhibition of cytoprotective autophagy, down-regulation of expression of anti-apoptotic protein survivin as well as suppression of AR- and AR-V7-dependent signaling (via down-regulation/degradation of both androgen receptors) were observed. Drug resistance remains a major challenge in prostate cancer treatment. Up to 30% of patients with CRPC are preliminary resistant to the first line drugs and basically all the patients over time eventually develop drug resistance to applied therapeutics [41]. Up-regulated autophagy was found in a number of prostate cancer tumors and therefore is considered to be an important resistance mechanism to chemo- and radiotherapies in CRPC [23]. Expression of AR-V7 has recently emerged to be an important mechanism of resistance to androgen receptor targeting therapies such as abiraterone and enzalutamide [27,42]. Thus, novel therapeutics which are able to overcome drug resistance and reveal cytotoxic activity at the same time are of high value and interest. It is important to note that no correlation of the drug-sensitivity and the cellular AR status has been observed; e.g., 22Rv1 (AR^+^, AR-V7^+^) and DU145 (AR^−^, AR-V7^−^) cells were more sensitive to the tested compounds in comparison with VCaP (AR^+^, AR-V7^+^) and PC-3 (AR^−^, AR-V7^−^) cells (Figure 1D), whereas LNCaP cells (AR^+^, AR-V7^−^, hormone therapy sensitive) exhibited an average sensitivity level. Thus, hormone therapy resistant prostate cancer cells exhibit no cross-resistance to the investigated conjugated.

Further examinations on the mode of action were performed for conjugate **11** using global proteome analysis. Conjugate **11** was chosen because it was the most promising out of the synthesized compounds based on the results of cytotoxicity and selectivity profile analyses. Using consequent bioinformatical analysis we were able to predict and further validate an activation of pro-apoptotic MAPK, namely p38 and JNK1/2 as well as Akt, while ERK1/2, playing an important role in a number of pro-survival processes was suppressed. This may further contribute to the ability of the substance to overcome drug resistance. Further analysis of proteomics data suggested treatment-mediated alteration of oxidative phosphorylation, which takes place in the inner mitochondrial membrane. Indeed, we were able to further confirm the targeting of mitochondria of the cancer cells and showed an elevation of the cytotoxic ROS levels as well as release of the cytotoxic mitochondrial proteins AIF and cytochrome C to cytoplasm. Simultaneously, we observed an increased Bax/Bcl-2 ratio, which is known to lead to mitochondrial dysfunction, followed by cytochrome C release and caspases activation, both ultimately leading to apoptosis (reviewed in [43]). Importantly, the drop down of the mitochondrial inner membrane potential (Δψ_m_), ROS up-regulation and cleavage of caspase-9 were observed after only 2 h following the treatment. Caspase-9 is an initiator caspase, which is activated (cleaved) in mitochondria-mediated apoptosis and later leads to the activation of effector caspase-3 (reviewed in [43]). The cleavage of caspase-9 prior to caspase-3 proves a canonical character of the caspases activation by the synthesized compounds and confirms that the activation is not an unspecific event resulting from cell death. Finally, mitochondrial targeting by conjugates **7** and **11** also explains the difference in IC_50_s generated with MTT and trypan blue exclusion assays for these compounds. The readout of MTT assay depends on the mitochondrial activity, and disruption/suppression of this activity prior to the cellular membrane rupture may therefore be detected by MTT assay, but not by trypan blue exclusion assay. Remarkably, cytotoxic activity of the compounds was also observed after 2 h treatment with the drugs in 22Rv1 cells. However, IC_50_ values were distinctly higher than after 48 h (IC_50_ = 24.2 µM, 92.39 µM, 20.45 µM, and 94.85 µM for conjugated **7**, **8**, **11**, and **12**, respectively, after 2 h or treatment). Thus, following the 2 h treatment the cytotoxic events has been already initiated but yet not completely exerted. This is in line with the detection of delayed effector caspase-3 activation, which occurs after longer treatment time (>2 h).

Anticancer drugs specifically targeting mitochondria attracted attention in the last 10 years and attempts have been made in order to introduce mitochondrial targeting moieties into existing drugs [44]. Remarkably, these drugs were reported to be more effective than their parental molecules (e.g., fluorouracil conjugated with selective mitochondria-localized moiety F16 [45]) and some were reported to be able to overcome drug resistance [44,46]. Recently, it has been demonstrated that oxidative phosphorylation is up-regulated in different cancer entities, including prostate cancer (specifically in primary tumors) [32,37]. Moreover, its correlation with hormone resistance of prostate cancer cells has been reported (reviewed in [47]). Consequently, the interest to inhibitors of oxidative phosphorylation, as a novel emerging target, has greatly increased [32]. During glycolysis the glucose molecule breaks down to pyruvate, which is later transported to mitochondria and further oxidized in Krebs cycle. Thus, inside of a living cell the quinone-conjugated glucose residue may be converted to pyruvate which can be further specifically delivered to mitochondria. Therefore, mitochondria targeting by the synthesized compounds could be promoted by their glucose-conjugate nature. Of note, the non-glycoside bond in these molecules cannot be easily hydrolyzed by the cytoplasmatic enzymes, hence the “warhead” quinone moieties may enter mitochondria still being glucose/pyruvate-conjugated which again contributes to their specificity.

## 4. Materials and Methods

### 4.1. Reagents and Antibodies

The investigated compounds have been synthesized as described in Supplementary information. The purity of the individual compound was verified by TLC, ^1^H- and ^13^C-NMR spectroscopy (Table S2). For details of synthesis, purification and structure elucidation see the Supplementary Information. Other reagents are listed in the Supplementary Information. Antibodies used are listed in Table S3.

### 4.2. Cell Lines and Culture Conditions

For general experiments human prostate cancer cell lines PC-3 and DU145 (docetaxel-resistant, androgen-independent, AR-FL(−), AR-V7(−)), 22Rv1 and VCaP (docetaxel-sensitive, androgen-independent, AR-FL(+), AR-V7(+), and LNCaP (docetaxel sensitive, androgen-dependent, AR-FL(+), AR-V7(−) were used [18,19,20]. PC-3, 22Rv1, LNCaP cells as well as the human prostate non-cancer cells PNT2 and RWPE-1 were purchased from ATCC (Manassas, VA, USA). VCaP were purchased from ECACC (Salisbury, UK). The human fibroblast cell line MRC-9, the human embryonic kidney cell line HEK 293T, as well as the human umbilical vascular endothelial cell line HUVEC were kindly donated by Prof. Dr. Dr. med. Sonja Loges (University Medical Center Hamburg-Eppendorf, Hamburg, Germany). Cells were cultured according to the manufacturers’ instructions and were recently authenticated by a commercial service (Multiplexion, Heidelberg, Germany). Culture conditions are described in the Supplementary Information.

### 4.3. In Vitro MTT-Based Drug Sensitivity Assay

MTT (3-(4,5-dimethylthiazol-2-yl)-2,5-diphenyltetrazolium bromide) cytotoxicity assay was used for the evaluation of cytotoxicity of the compounds or their combinations. Experiments were performed as previously described [48]. In brief, 6000 cells/well were seeded in 96-well plate, incubated overnight, and treated with the drugs in 100 µL/well of 10% FBS/RPMI media containing 2 g/L of D-(+)-glucose for 48 h, unless otherwise stated. For 2 h treatment the cells were treated with the drugs for 2 h in the culture media, then the media was exchanged to fresh drug-free media (100 µL/well) containing MTT reagent and the further steps were performed as previously described [48].

### 4.4. In Vitro Trypan Blue-Based Viability Assay

The *in vitro* effect of the drugs on cell viability and proliferation was evaluated using trypan blue exclusion assay as described before with minor modifications [49]. In brief, cells (0.2 × 10^6^ cells/well) were seeded in 12-well plates, incubated overnight, the media was replaced with fresh media (1 mL/well) and treated with the drugs for 48 h. Cells were harvested by trypsination and the viability was measured using trypan blue staining and Beckman Coulter Vi-CELL (Beckman Coulter, Krefeld, Germany).

### 4.5. ELISA

To determine the expression of GLUT-1 in the ten non-treated cell lines the ELISA kit for Glucose transporter-1 (#SEB184Hu, Cloud-Clone Corp., Houston, TX, USA) was used. Human prostate cancer cells (PC-3, DU145, 22Rv1, VCaP, and LNCaP) and human non-cancer cells (MRC-9, HUVEC, HEK 293, PNT2, and RWPE-1) were seeded in the ø 6 cm TC dishes (1 × 10^6^ cells/well in 5 mL/dish of culture media), incubated overnight and then harvested with scratching, resuspended in Western blotting lysis buffer and proceeded according to the manufacture’s protocols. For this assay 30 µg/well of total protein in 100 µL/well were loaded.

### 4.6. Glucose Uptake Assay

The effects of the compounds on glucose uptake were determined by Glucose Uptake Cell-Based Assay Kit (Cayman chemicals, Ann Arbor, MI, USA). PC-3 or 22Rv1 cells (100,000 cells/well) were seeded into 12-well plates, incubated overnight and treated with the investigated drugs in 500 µL/well of glucose-free 0.1% FBS/RPMI media for 24 h. Then, the 50 µL/well of 2-NBDG solution (400 µg/mL) in glucose-free 0.1% FBS/RPMI media were added to each well (final concentration of 2-NBDG in wells was 40 µg/mL). After 2 h of incubation the cells were harvested by trypsination, washed with PBS and resuspended in 100 µL/sample of PI/PBS solution (1 µg/mL). Following 10 min incubation, 300 µL/sample of PBS were added and the glucose uptake in viable (PI-negative) cells was evaluated using flow cytometry technique and quantified using BD Bioscience Cell Quest Pro v.5.2.1. software.

### 4.7. Colony Formation Assay

Colony formation assays were performed as described before [49]. In brief, cells were treated with the drugs for 48 h and then 100 alive cells were plated into each well of 6-well plates and incubated for 10 days. Surviving colonies were fixed, stained with Giemsa solution and counted.

### 4.8. Cell Cycle and DNA Fragmentation Analysis

The effect on cell cycle progression was analyzed by flow cytometry technique using PI staining as reported before [48]. In brief, cells were pre-incubated overnight in 6-well plates (0.2 × 10^6^ cells/well in 2 mL/well), treated with the drugs for 48 h. Then cells were harvested, fixed, stained with PI, and analyzed using FACS Calibur (BD Bioscience, San Jose, CA, USA) and BD Bioscience Cell Quest Pro v.5.2.1. software (BD Bioscience).

### 4.9. Detection of Apoptotic Cells by Annexin-V-FITC/PI Double Staining

Induction of apoptosis was analyzed by flow cytometry technique using annexin-V-FITC and propidium iodide (PI) double staining. The experiment was performed as previously described [16]. In brief, cells were seeded and treated as described for the cell cycle analysis, cells were harvested by trypsination, stained, and analyzed using a FACS Calibur machine and BD Cell Quest Pro software.

### 4.10. Protein Preparation and Western Blotting

Preparation of protein extracts for western blotting was performed as described previously [20,50]. In brief, for western blotting, 1 × 10^6^ cells/well were seeded in Petri dishes (ø 6 cm TC Dish (Sarstedt, Numbrecht, Germany) in 5 mL/dish), incubated overnight and treated with drugs in 5 mL/dish for an indicated time. Cells were harvested using a cell scraper, pelleted, and resuspended in the lysis buffer containing protease and phosphatase inhibitors. Further sample preparation and western blotting were performed as previously described with slight modifications [20,50]. Relative optical density of the detected bands was quantified with Quantity One 4.6 software (Bio-Rad, Hercules, CA, USA). The primary and secondary antibodies used are listed in the Supplementary Information.

### 4.11. Proteomic Analysis of the Differentially Expressed Proteins

22Rv1 cells were seeded in T75 culture bottles (4 × 10^6^ cells/bottle in 20 mL/bottle), treated for 48 h with the 5 µM of compound 11 or vehicle (DMSO), harvested by scratching, washed twice with ice-cold PBS and flash-frozen. Cells were lysed and sonicated. The protein extracts were then heat-denaturated, reduced, alkylated and digested by trypsin. Peptides were chromatographically separated using full recovery autosampler vials and a nano-UPLC system (Acquity, Waters Corporation, Milford, MS, USA), coupled via electrospray ionization (ESI) to a tandem mass spectrometer equipped with a quadrupole and an orbitrap (QExactive, Thermo Fisher Scientific, Bremen, Germany), using MS/MS mode in data dependent acquisition (DDA) and data independent acquisition (DIA). The peptides, analyzed with DDA-LC-MS/MS, were identified with the search engine Sequest, integrated in the Proteome Discoverer software version 2.0 (Thermo Fischer Scientific) using the human Uniprot protein database (EMBL; Hinxton Cambridge, UK, release December 2018). Proteins were considered to be significantly regulated if 1.5 ≤ fold change ≤ 1/1.5 and *p* ≤ 0.05 (Student’s *t*-test). For the details of sample preparation and proceeding, data acquisition and analysis see the Supplementary information.

### 4.12. Bioinformatics Analysis of Differentially Expressed Proteins

Network analyses and gene ontology classification were performed using the Ingenuity Pathways Analysis (IPA) (QIAGEN Bioinformatics, Venlo, The Netherlands, https://www.qiagenbioinformatics.com/products/ingenuity-pathway-analysis/) algorithms. The list of significantly regulated proteins (1.5 ≤ fold change ≤ 1/1.5; *p* ≤ 0.05) discovered by global proteome analysis were inserted into the IPA software to explore the gene ontology, protein interaction networks as well as relevant biological molecules and processes affected by the drug. *z*-Score algorithm analysis was used to identify biological functions which are expected to be activated (*z*-score > 0) or suppressed (*z*-score < 0) in the cells upon treatment. The *p*-values were calculated with the Fischer’s exact test by IPA software. The shortest hypothetical pathway network was constructed, showing the most relevant direct and indirect connections of proteins found to be regulated by the compound.

### 4.13. Evaluation of Intracellular ROS

Intracellular ROS levels were estimated using the ROS-sensitive CM-H_2_DCFDA reagent (Cat. No. C6827, Molecular probes, Invitrogen, Eugene, OR, USA) as described before [51]. In brief, 22Rv1 cells were seeded in 12-well plates (0.2 × 10^6^ cells/well in 1 mL/well of media) and incubated overnight. The media was changed into freshly made pre-warmed 4 µM CM-H_2_DCFDA/PBS (0.5 mL/well) and incubated for 30 min (37 °C, 5% CO_2_, in the dark). Then, the CM-H_2_DCFDA solution was exchanged with 1 mL/well of pre-warmed PBS containing the drugs or H_2_O_2_ (positive control) at the indicated concentrations. After 2 h of incubation the cells were trypsinized and immediately analyzed by flow cytometry technique according to the manufacture’s protocols.

### 4.14. Mitochondrial Membrane Potential (Δψ) Assay

The loss of mitochondrial membrane potential (Δψ) was measured by flow cytometry technique using staining with Δψ-sensitive dye JC-1 (5,5′,6,6′-tetrachloro-1,1′,3,3′-tetraethylimidacarbocyanine iodide). Cells were seeded in 12-well plates (100 × 10^3^ cells/well in 1 mL/well) and incubated overnight. Then, the media was changed to PBS (1 mL/well) containing the investigational drugs. After 2 h of incubation (37 °C, 5% CO_2_, in the dark) the cells were trypsinized, pelleted, resuspended in 100 µL of 2 µM JC-1/PBS, incubated for 1 h (37 °C, 5% CO_2_, in the dark) and Δψ loss was measured by flow cytometry technique.

### 4.15. Cell Fractionation

22Rv1 cells were seeded in T75 culture bottles (4 × 10^6^ cells/bottle in 20 mL/bottle), treated for 48 h and harvested by scratching. The further separation of cytosolic, mitochondrial and nuclear fractions was performed using the Cell Fractionation Kit ab109719 (abcam, Cambridge, MA) as previously described [51]. Mitochondrial and cytosolic fractions were concentrated using Amicon^®^ Ultra-2 Centrifugal Filter device (Cat. No. UFC203024, Merck, Darmstadt, Germany). Nuclear fractions were additionally homogenized using QIAshedder kit (QIAGEN, Hilden, Germany).

### 4.16. Statistical Analysis

Statistical analyses were performed using GraphPad Prism software v. 5.01 (GraphPad Prism software Inc., La Jolla, CA, USA). Data are presented as mean ± SEM (standard error of the mean). All the experiments were performed in triplicates and repeated at least three times. The unpaired Student’s t-test was used for comparison of two groups. The one-way analysis of variance (ANOVA), followed by a post-hoc Dunnett’s test was used for multiple groups comparisons. Differences were considered to be statistically significant if *p* < 0.05.

## 5. Conclusions

In conclusion, we found natural 1,4-naphtoquinone derivatives with promising anti-cancer activity in human drug-resistant prostate cancer cells and synthesized new glucose derivatives (6-O-(1,4-naphtaquinone-2-yl)-D-glucose conjugates) with improved selectivity. The compounds exhibited selective cytotoxicity in human prostate cancer cells due to a higher uptake rate, which most likely results from the Warburg effect. The glucose conjugates were able to overcome cancer cell drug resistance and suppress pro-survival mechanisms. Cytotoxic activity is executed via primary intracellular targeting of cancer cell mitochondria leading to cytotoxic ROS production as well as to AIF and cytochrome C release to cytoplasm, which results in caspase-9 and -3 activation and ultimately leads to apoptotic death of cancer cells (Figure 5I). Our simple and effective way of total synthesis of these compounds enables us to produce amounts necessary for further *in vivo* testing and clinical trials.

## Supplementary Materials

The following are available online at https://www.mdpi.com/2072-6694/11/11/1690/s1; Chemistry, Biology and Methods of Proteomics; Table S1: Starting compounds used for the synthesis, Table S2: Synthesized compounds; Reagents and antibodies, Table S3: List of antibodies used, Table S4: Cytotoxic activity of the synthesized compounds, Table S5: The regulated proteins discovered by proteome analysis, Figure S1: Original files for Figure 3F,G (Western blotting); Figure S2: Original files for Figure 4E (Western blotting); Figure S3: Original files for Figure 5F (Western blotting); Figure S4: Original files for Figure 5G (Western blotting); Figure S5: Original files for Figure 5H (Western blotting).

## Abbreviations

2-NBDG	2-deoxy-2-((7-nitro-2,1,3-benzoxadiazol-4-yl)amino)-D-glucose
ΔΨm	mitochondrial membrane potential (MMP)
AIF	apoptosis inducing factor
Apig	apigenin
Aniso	anisomycin
AR	androgen receptor
AR-V7	androgen receptor splice variant 7
CCCP	carbonyl cyanide 3-chlorophenylhydrazone
Ccl-B	cytochalasin B
CRPC	castration-resistant prostate cancer
Glc	glucose
GLUT-1	glucose transporter-1
MTT	3-(4,5-dimethylthiazol-2-yl)-2,5-diphenyltetrazolium bromide
NaC	N-acetyl-L-cysteine
Plt	phloretin
ROS	reactive oxygen species
RT	room temperature
SI	selectivity index
