# Supplementary material for: Successful Targeting of the Warburg Effect in Prostate Cancer by Glucose-Conjugated 1,4-Naphthoquinones
Source: Cancers (Basel). 2019 Oct 30;11(11):1690. doi: 10.3390/cancers11111690 (PMC6896172; doi:10.3390/cancers11111690)
Supplement: Supplementary file 1 [file cancers-11-01690-s001.zip › cancers-634962-supplementary/cancers-634962 - supplementary final/cancers-634962 - supplementary final.pdf]

Supplementary Materials

Successful Targeting of the Warburg Effect in Prostate Cancer by Glucose-Conjugated 1,4-Naphthoquinones

Sergey A. Dyshlovoy, Dmitry N. Pelageev, Jessica Hauschild, Ksenia L. Borisova, Moritz Kaune, Christoph Krisp, Simone Venz, Yurii E. Sabutskii, Ekaterina A. Khmelevskaya, Tobias Busenbender, Vladimir A. Denisenko, Natalia D. Pokhilo, Lyubov N. Atopkina, Markus Graefen, Hartmut Schlüter, Valentin A. Stonik, Carsten Bokemeyer, Victor Ph. Anufriev and Gunhild von Amsberg

Chemistry

General Synthetic Procedures and Methods

All melting points were determined with a Boetius apparatus (Dresden, Germany) and were uncorrected. Analytical grade chemicals and solvents were used. The ^{1}H and ^{13}C NMR spectra were recorded using Bruker Avance-300 (300 MHz), Bruker Avance III-500 HD (500 MHz) and Bruker Avance III-700 (700 MHz) spectrometers (Bruker Corporation, Germany) using $CDCl_3$, DMSO-d_6 and DMSO-d_6-D_2O (10:1, v/v) as the solvents with the signal of the residual nondeuteriated solvent as the internal reference. The IR absorption spectra were recorded using an IR-FT spectrophotometer Bruker Equinox 55. Mass spectra were recorded using an AMD 604S spectrometer (AMDIntectra, Harpstedt, Germany), direct sample inlet, ionizing energy of 70 eV, and elevated temperature and Agilent 6510 Q-TOF LC mass spectrometer (Agilent, Santa Clara, CA, USA) (electrospray ionization, positive mode). The course of reactions was monitored, and the purity of the compounds obtained was assessed by TLC. Merck Kieselgel 60F-254 plates (Kenilworth, NJ, USA) were preliminarily treated with 0.05 M tartaric acid in MeOH and dried at ~50°C for 2-3 h; a 3:1 hexane/acetone mixture was used as an eluent. Preparative column chromatography was performed on silica gel Alfa Aesar 40/65 µm (Ward Hill, MA, USA), using *n*-hexane-acetone. The yields were not optimized.

Lawsone, juglone and naphthazarin were purchased from Sigma-Aldrich (Munich, Germany). Compounds **5a, 9a, 13a, 17a** and **25a** were synthesized as described before (see Table S1). Compound **21a** was synthesized from 7-chloro-6-ethyl-5,8-dihydroxy-2-methoxy-1,4-naphthoquinone **21**. Compounds **1, 5, 9, 13, 17** and **25** were synthesized by methylation of appropriate compounds **5a, 9a, 13a, 17a and 25a** with CH_2N_2 as described before (see Table S1). Compound **21** is the side product of echinochrome A synthesis (ref. see Table S1). 6-Deoxy-6-iodo-1,2:3,5-di-O-isopropylidene-α-D-glucofuranose was synthesized from 1,2:3,5-di-O-isopropylidene-6-O-tosyl-α-D-glucofuranose.

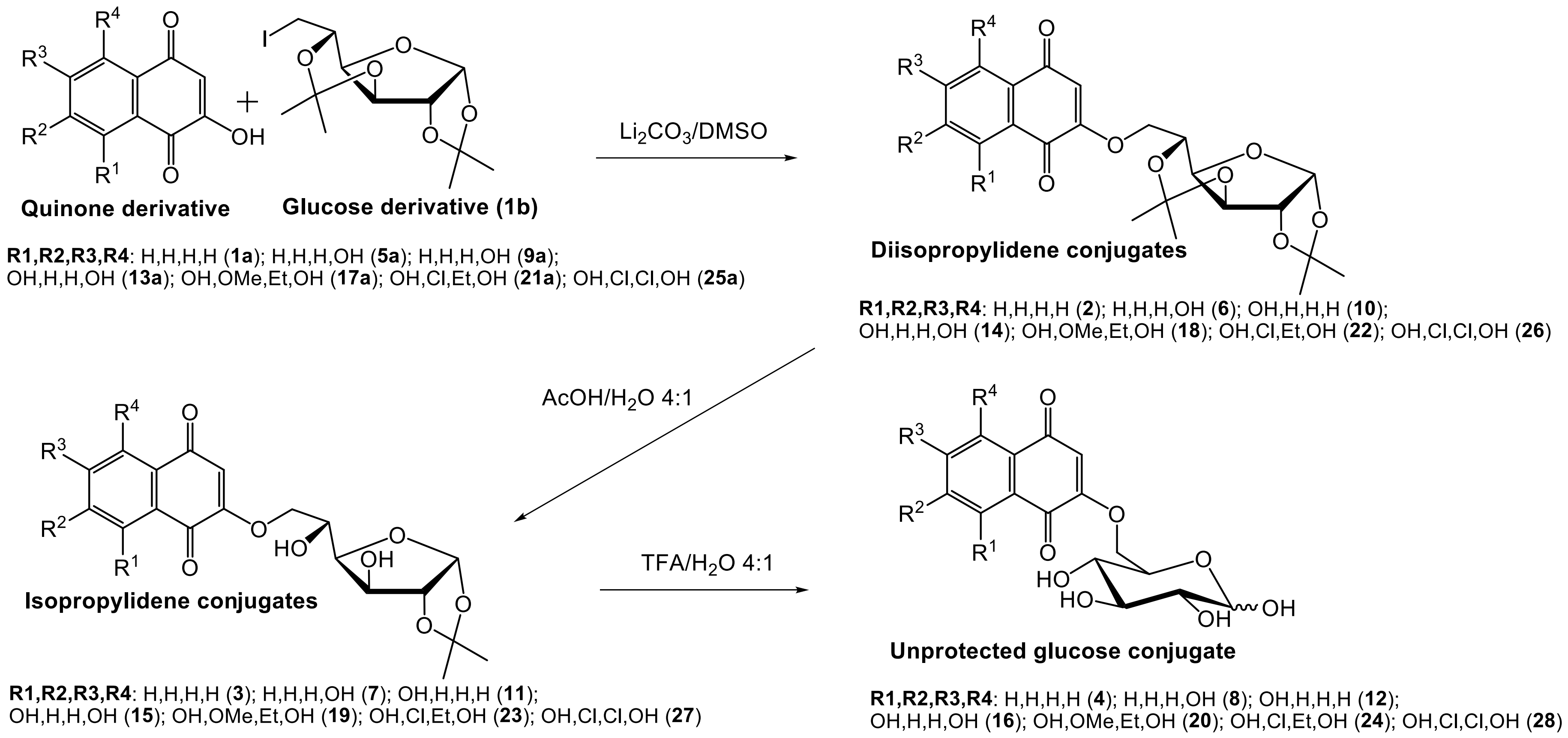

Quinone derivative
Glucose derivative (1b)
R1,R2,R3,R4: H,H,H,H (1a); H,H,H,OH (5a); H,H,H,OH (9a); OH,H,H,OH (13a); OH,OMe,Et,OH (17a); OH,Cl,Et,OH (21a); OH,Cl,Cl,OH (25a)
Li2CO3/DMSO
Diisopropylidene conjugates
R1,R2,R3,R4: H,H,H,H (2); H,H,H,OH (6); OH,H,H,H (10); OH,H,H,OH (14); OH,OMe,Et,OH (18); OH,Cl,Et,OH (22); OH,Cl,Cl,OH (26)
AcOH/H2O 4:1
Isopropylidene conjugates
R1,R2,R3,R4: H,H,H,H (3); H,H,H,OH (7); OH,H,H,H (11); OH,H,H,OH (15); OH,OMe,Et,OH (19); OH,Cl,Et,OH (23); OH,Cl,Cl,OH (27)
TFA/H2O 4:1
Unprotected glucose conjugate
R1,R2,R3,R4: H,H,H,H (4); H,H,H,OH (8); OH,H,H,H (12); OH,H,H,OH (16); OH,OMe,Et,OH (20); OH,Cl,Et,OH (24); OH,Cl,Cl,OH (28)

Table S1. Starting compounds used for the synthesis.

No	Formula	Spectral Information, References, Notes
**1a**	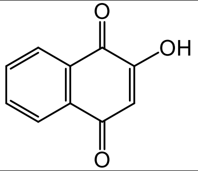	**2-hydroxy-1,4-naphthoquinone (1a):**<br>purchased from Sigma-Aldrich (Munich, Germany)
**5a**	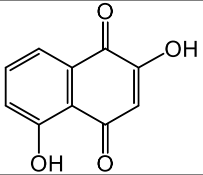	**2,5-dihydroxy-1,4-naphthoquinone (5a):**<br>was synthesized as described in Fieser L.F., Dunn J.T. The addition of dienes to halogenated and hydroxylated naphthoquinones // *J Am Chem Soc*, **1937**, *59*, 1016-1021.
**9a**	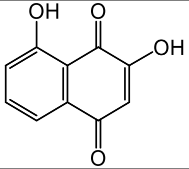	**2,8-dihydroxy-1,4-naphthoquinone (9a):**<br>was synthesized as described in Fieser L.F., Dunn J.T. The addition of dienes to halogenated and hydroxylated naphthoquinones // *J Am Chem Soc*, **1937**, *59*, 1016-1021.
**13a**	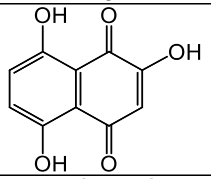	**2,5,8-trihydroxy-1,4-naphthoquinone (13a):**<br>was synthesized as described in Yakubovskaya A.Y., Pokhilo N.D., Anufriev V.Ph., Anisimov M.M. Synthesis and antimicrobial and antifungal activities of compounds of the naphthazarin series // *Pharm Chem J*, **2009**, *43*, 396-398.
**17a**	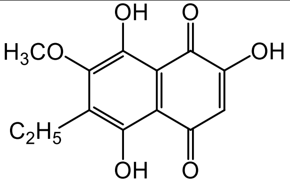	**6-ethyl-2,5,8-trihydroxy-7-methoxy-1,4-naphthoquinone (17a):**<br>was synthesized as described in Glazunov V.P., Tchizhova A.Ya., Pokhilo N.D., Anufriev V.Ph., Elyakov G.B. First direct observation of tautomerism of monohydroxynaphthazarins by IR-spectroscopy // *Tetrahedron*, **2002**, *58*, 1751-1757.
**21a**	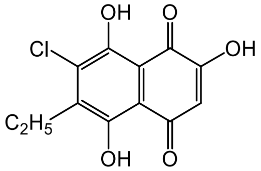	**7-chloro-6-ethyl-2,5,8-trihydroxy-1,4-naphthoquinone (21a):**<br>red solid;<br>yield 700 mg, 87%;<br>mp 150-155°C;<br>**IR ($CHCl_3$) ν_{max}:** 3416, 2979, 2940, 2880, 1660, 1624, 1612, 1602, 1558, 1428, 1395, 1355, 1335, 1299, 1270 cm^{-1};<br>**^{1}H NMR ($CDCl_3$, 500 MHz) δ:** 1.21 (t, *J* = 7.5, 3H, $C\underline{H_3}CH_2$), 2.96 (q, *J* = 7.5, 2H, $CH_3C\underline{H_2}$), 6.39 (s, 1H, H_3), 7.38 (br s, 1H, C(2)-OH), 12.13 (s, 1H, C(8)-OH), 13.29 (s, 1H, C(5)-OH);<br>**^{13}C NMR (125 MHz, $CDCl_3$) δ:** 12.1 ($\underline{C}H_3CH_2$), 21.6 ($CH_3\underline{C}H_2$), 108.4 (C4a), 109.1 (C8a), 111.5 (C3), 131.6 (C7), 147.4 (C6), 156.5 (C8), 156.8 (C2), 158.1 (C5), 179.7 (C1), 186.7 (C4);<br>**HRMS (EI):** *m/z* $[M]^+$ calcd for $C_{12}H_9ClO_5$: 268,0136; found: 268,0129.

**25a**	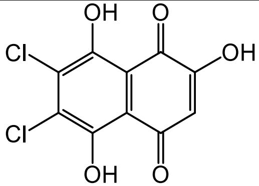	**6,7-dichloro-2,5,8-trihydroxy-1,4-naphthoquinone (25a)**<br>was synthesized as described in Anufriev V.Ph., Novikov V.L., Maximov O.B., Elyakov G.B., Levitsky D.O., Lebedev A.V., Sadretdinov S.M., Shvilkin A.V., Afonskaya N.I., Ruda M.Ya., Cherpachenko N.M. Synthesis of some hydroxynaphthazarins and their cardioprotective effects under ischemia-reperfusion *in vivo* // *Bioorg. Med. Chem. Lett.*, **1998**, *8*, 587-592.
	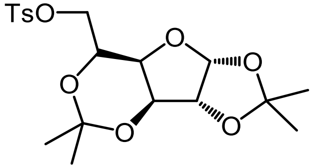	**1,2:3,5-di-O-isopropylidene-6-O-tosyl-α-D-glucofuranose**<br>was synthesized as described in Bruce Ronsen, Sudershan K. Arora, Albert Varghese Thomas Derivatives of alpha-D-glucofuranose and intermediates for preparing these derivatives European Patent 0379397A2.
**1b**	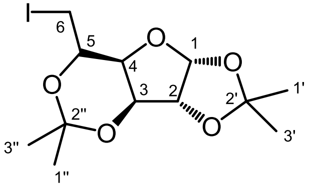	**6-deoxy-6-iodo-1,2:3,5-di-O-isopropylidene-α-D-glucofuranose:**<br>mp 61–63 °C; (**lit.** mp 63.0–63.5 °C).<br>**^{1}H NMR (CDCl$_3$, 500 MHz) *δ*:** 1.33 (s, 3H, C(1')H$_3$), 1.37 (s, 3H, C(1'')H$_3$), 1.38 (s, 3H, C(3'')H$_3$), 1.49 (s, 3H, C(3')H$_3$), 3.20 (dd, *J* = 10.5, 8.0 Hz, 1H, H$_{6a}$), 3.46 (dd, *J* = 10.5, 3.3 Hz, 1H, H$_{6b}$), 3.54 (m, 1H, H$_5$), 4.23 (m, 2H, H$_3$, H$_4$), 4.58 (d, *J* = 3.7 Hz, 1H, H$_2$), 5.98 (d, *J* = 3.7 Hz, 1H, H$_1$);<br>**^{13}C NMR (125 MHz, CDCl$_3$) δ:** 6.5 (C-6), 23.8 (C-1''), 24.0 (C-3''), 26.5 (C-1'), 27.2 (C-3'), 72.0 (C-5), 75.0 (C-3), 82.9 (C-4), 83.9 (C-2), 101.4 (C-2''), 106.3 (C-1), 112.3 (C-2');<br>**lit.: CA 58, 11456a.** Petrov K.A., Nifant'ev E.E., Shchegolev A.A., Terekhov V.G. Synthesis and alkylation of phosphites and phosphinites of 1,2:3,4-di-O-isopropylidene-α-D-galactopyranose and 1,2:3,5-di-O-isopropylidene-α-D-glucofuranose // *Zhurnal Obshchei Khimii*, **1964**, *34*, 1459-1462.

Procedures to Prepare 6-Deoxy-6-Iodo-1,2:3,5-di-O-Isopropylidene-α-D-Glucofuranose

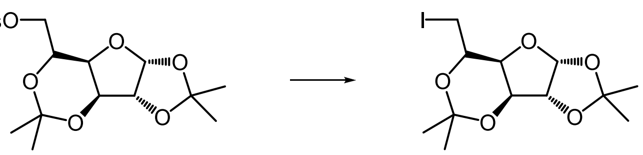

A mixture of 1,2:3,5-di-O-isopropylidene-6-O-tosyl-α-D-glucofuranose (4.14 g, 10 mmol) and sodium iodide (7.5 g, 50 mmol) in methyl ethyl ketone (150 mL) was refluxed for 4 hours. The precipitate was filtered, washed with methyl ethyl ketone. The combined filtrate was evaporated under reduced pressure, dissolved in chloroform. The precipitate of sodium iodide was filtered, washed with chloroform. The filtrate was evaporated under reduced pressure, the residue was purified by column chromatography (SiO_2, hexane-acetone, 100: 1) to give 3.1 g (84%) of 6-deoxy-6-iodo-1,2:3,5-di-O-isopropylidene-α-D-glucofuranose.

*Procedures to Prepare 7-Chloro-6-Ethyl-2,5,8-Trihydroxy-1,4-Naphthoquinone (**21a**)*

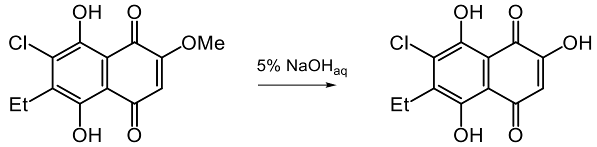

A solution of 7-chloro-6-ethyl-5,8-dihydroxy-2-methoxy-1,4-naphthoquinone **21** (848 mg, 3 mmol) in aqueous solution of sodium hydroxide (50 mL) was refluxed for 2 hours. The reaction mixture was cooled, acidified with hydrochloric acid. The precipitate was filtered off and dried. The filtrate was extracted with ethyl acetate, dried with anhydrous Na_2SO_4, and evaporated under reduced pressure. The residue and the precipitate were combined, purified by column chromatography (SiO_2, hexane-acetone, 10:1) to give 7-chloro-6-ethyl-2,5,8-trihydroxy-1,4-naphthoquinone (**21a**) 700 mg (87%).

*General Procedures to Prepare Diisopropylidene Conjugates (**2, 6, 10, 14, 18, 22, 26**)*

A mixture of 2-hydroxy-1,4-naphthoquinone **1a**, **5a**, **9a**, **13a**, **17a**, **21a** or **25a** (2 mmol), 6-deoxy-6-iodo-1,2:3,5-di-O-isopropylidene-α-D-glucofuranose (1.85 g, 5 mmol) and lithium carbonate (148 mg, 2 mmol) in 10 ml of DMSO was heated in an oil bath at 70 °C for 24 hours. Then the mixture was poured into water (200 ml) and extracted with diethyl ether. The ether extract was dried with anhydrous Na_2SO_4, concentrated under reduced pressure, and separated by column chromatography (SiO_2, eluted with hexane-acetone 50:1) to give unreacted 6-deoxy-6-iodo-1,2:3,5-di-O-isopropylidene-α-D-glucofuranose and conjugate **2**, **6**, **10**, **14**, **18**, **22** or **26**. The aqueous layer was acidified, extracted with ethyl acetate, and separated by column chromatography (SiO_2 acidified with HCl, eluted with hexane-acetone 20:1) to give unreacted 2-hydroxy-1,4-naphthoquinone **1a, 5a, 9a, 13a, 17a, 21a** or **25a**.

*General Procedures to Prepare Isopropylidene Conjugates (**3, 7, 11, 15, 19, 23, 27**)*

Conjugates **2, 6, 10, 14, 18, 22** or **26** (0.25 mmol) were dissolved in 80% acetic acid (10 mL) and left for 24 hours. The solutions were evaporated under reduced pressure, the residues were separated by column chromatography (SiO_2, eluted with hexane-acetone 10:1) which resulted in conjugates **3, 7, 11, 15, 19, 23** or **27**.

*General Procedures to Prepare Deprotected Conjugates (**4, 8, 12, 16, 20, 24, 28**)*

Conjugates **3**, **7**, **11**, **15**, **19**, **23** or **27** (0.1 mmol) were dissolved in a mixture trifluoroacetic acid-water (4:1 mL) and left for 2 hours. The solutions were evaporated under reduced pressure, the residues were separated by column chromatography (SiO_2, eluted with hexane-acetone, 3:1) which resulted in conjugates **4, 8, 12, 16, 20, 24** or **28**.

Table S2. Synthesized compounds.

No	Formula	Spectral Information, References, Notes
1	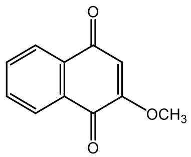	**2-Methoxy-1,4-naphthoquinone (1):**<br>pale yellow solid;<br>mp 186–188 °C (lit. 187.0–187.8 °C).<br>**^{1}H NMR ($CDCl_3$, 500 MHz) δ:** 3.89 (s, 3H, CH_3), 6.16 (s, 1H, H_3), 7.70 (dt, *J* = 1.3, 7.5 Hz, 1H, H_7), 7.74 (dt, *J* = 1.3, 7.5 Hz, 1H, H_6), 8.07 (dd, *J* = 1.2, 7.5 Hz, 1H, H_8), 8.11 (dd, *J* = 1.2, 7.5 Hz, 1H, H_5);<br>**lit.:** Shao-Hung Wang, Chih-Yu Lo, Zhong-Heng Gwo, Hong-Jhih Lin, Lih-Geeng Chen, Cheng-Deng Kuo, Jin-Yi Wu // Molecules, **2015**, *20*, 11994-12015;<br>doi:10.3390/molecules200711994
2	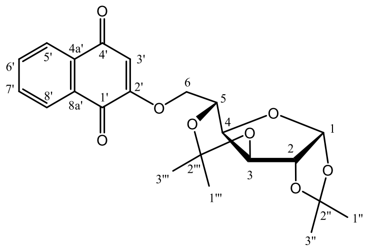	**1,2:3,5-di-O-isopropylidene-6-O-(1,4-naphthoquinone-2-yl)-α-D-glucofuranose (2):**<br>pale yellow solid;<br>yield 293 mg, 55% (recovery of 2-hydroxy-1,4-naphthoquinone **1a** 125 mg);<br>mp 127-129°C;<br>**IR ($CHCl_3$) ν_{max}:** 2994, 2941, 1688, 1655, 1610, 1580, 1456, 1384, 1377, 1331, 1263, 1244 cm^{-1};<br>**^{1}H NMR ($CDCl_3$, 300 MHz) *δ*:** 1.32 (s, 3H, C(1'')H_3), 1.37 (s, 3H, C(1''')H_3), 1.39 (s, 3H, C(3''')H_3), 1.49 (s, 3H, C(3'')H_3), 3.97 (ddd, *J* = 7.7, 6.4, 2.5 Hz, 1H, H_5), 4.13 (dd, *J* = 11.0, 6.4 Hz, 1H, H_{6a}), 4.21 (dd, *J* = 11.0, 2.5 Hz, 1H, H_{6b}), 4.30 (d, *J* = 3.9 Hz, 1H, H_3),4.49 (dd, *J* = 7.7, 3.9 Hz, 1H, H_4), 4.60 (d, *J* = 3.7 Hz, 1H, H_2), 6.01 (d, *J* = 3.7 Hz, 1H, H_1), 6.19 (s, 1H, $H_{3'}$), 7.66-7.76 (m, 2H, $H_{6'}$, $H_{7'}$), 8.02-8.12 (m, 2H, $H_{5'}$, $H_{8'}$);<br>**^{13}C NMR (75 MHz, $CDCl_3$) δ:** 23.8 (C-1'''), 24.1 (C-3'''), 26.5 (C-1''), 27.0 (C-3''), 69.4 (C-6), 70.3 (C-5), 75.1 (C-3), 78.9 (C-4), 84.1 (C-2), 101.4 (C-2'''), 106.5 (C-1), 110.7 (C-3′), 112.5 (C-2''), 126.1 (C-5′), 126.6 (C-8′), 131.2 (C-4a′), 132.0 (C-8a′), 133.4 (C-7′), 134.2 (C-6′), 159.6 (C-2′), 179.6 (C-1′), 184.9 (C-4′);<br>**HRMS (EI):** *m/z* [M]$^+$ calcd for $C_{22}H_{24}O_8$: 416.1471; found: 416.1463.
3	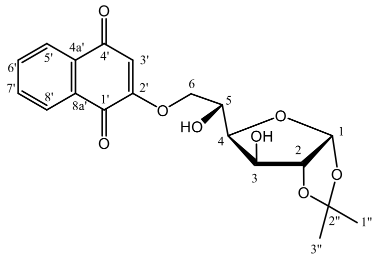	**1,2-O-isopropylidene-6-O-(1,4-naphthoquinone-2-yl)-α-D-glucofuranose (3):**<br>pale yellow solid;<br>yield 89 mg (95%);<br>mp 134-137°C;<br>**IR ($CHCl_3$) ν_{max}:** 3460, 3056, 3006, 1677, 1656, 1615, 1580, 1562, 1455, 1385, 1376, 1330, 1307, 1264 cm^{-1};<br>**^{1}H NMR ($CDCl_3$, 700 MHz) *δ*:** 1.34 (s, 3H, C(1'')H_3), 1.50 (s, 3H, C(3'')H_3), 4.06 (dd, *J* = 9.8, 6.4 Hz, 1H, H_{6a}), 4.20 (dd, *J* = 7.7, 2.8 Hz, 1H, H_4), 4.30 (dd, *J* = 9.8, 2.3 Hz, 1H, H_{6b}), 4.48 (ddd, *J* = 7.7, 6.4, 2.3 Hz, 1H, H_5), 4.52 (d, *J* = 2.8 Hz, 1H, H_3), 4.70 (d, *J* = 3.6 Hz, 1H, H_2), 6.06 (d, *J* = 3.6 Hz, 1H, H_1), 6.19 (s, 1H, $C_{3'}$), 7.70 (dt, *J* = 7.6, 1.4 Hz, 1H, $H_{7'}$), 7.75 (dt, *J* = 7.6, 1.4 Hz, 1H, $H_{6'}$), 8.06 (dd, *J* = 7.6, 1.4 Hz, 1H, $H_{5'}$), 8.07 (dd, *J* = 7.6, 1.4 Hz, 1H, $H_{8'}$);<br>**^{13}C NMR (176 MHz, $CDCl_3$) δ:** 26.2 (C-1''), 26.8 (C-3′′), 67.8 (C-5), 71.9 (C-6), 75.6 (C-3), 79.8 (C-4), 85.2 (C-2), 105.1 (C-1), 110.7 (C-3′), 111.9 (C-2''), 126.4 (C-5′), 126.7 (C-8'), 130.7 (C-8a'), 132.1 (C-4a'), 133.4 (C-7'), 134.8 (C-6'), 159.0 (C-2'), 181.2 (C-1'), 184.3 (C-4');<br>**HRMS (EI):** *m/z* [M]$^+$ calcd for $C_{19}H_{20}O_8$: 376.1158; found: 376.1153.

**4**	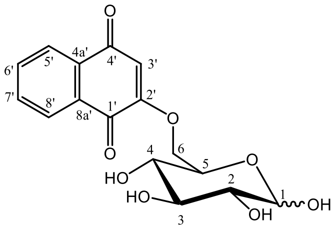	**6-O-(1,4-naphthoquinone-2-yl)-D-glucopyranose (4):**<br>pale yellow solid;<br>yield 27 mg (82%);<br>**^{1}H NMR (500 MHz, DMSO-d$_6$) *δ*:**<br>**carbohydrate moiety:**<br>***α*-anomer:** 3.19 (dd, *J* = 8.6, 3.4 Hz, 1H, H$_2$), 3.20 (t, *J* = 10.0 Hz, 1H, H$_4$), 3.50 (dd, *J* = 10.0, 8.6 Hz, 1H, H$_3$), 3.98 (ddd, *J* = 10.0, 5.6, 1.5 Hz, 1H, H$_5$), 4.16 (dd, *J* = 11.5, 5.6 Hz, 1H, H$_{6a}$), 4.22 (dd, *J* = 11.5, 1.5 Hz, 1H, H$_{6b}$), 4.96 (d, *J* = 3.4 Hz, 1H, H$_1$);<br>**β-anomer:** 2.97 (dd, *J* = 8.2, 7.7 Hz, 1H, H$_2$), 3.20 (m, 1H, H$_3$), 3.21 (m, 1H, H$_4$), 3.54 (ddd, *J* = 9.1, 5.8, 1.3 Hz, 1H, H$_5$), 4.14 (dd, *J* = 11.2, 5.8 Hz, 1H, H$_{6a}$), 4.26 (dd, *J* = 11.2, 1.3 Hz, 1H, H$_{6b}$), 4.39 (d, *J* = 7.7 Hz, 1H, H$_1$);<br>**naphthoquinone moiety:** 6.36 (s, 1H, H$_{3'}$), 7.82 (dt, *J* = 7.4, 1.6 Hz, 1H, H$_{6'}$), 7.85 (dt, *J* = 7.4, 1.6 Hz, 1H, H$_{7'}$), 7.96 (dd, *J* = 7.4, 1.6 Hz, 1H, H$_{5'}$), 8.00 (dd, *J* = 7.4, 1.6 Hz, 1H, H$_{8'}$).<br>**^{13}C NMR (125 MHz, DMSO-d$_6$) δ:**<br>**carbohydrate moiety:**<br>***α*-anomer:** 69.3 (C-5), 69.8 (C-6), 70.2 (C-4), 72.1 (C-2), 72.9 (C-3), 92.4 (C-1);<br>**β-anomer:** 69.6 (C-6), 69.9 (C-4), 73.6 (C-5), 74.6 (C-2), 76.4 (C-3), 97.0 (C-1);<br>**naphthoquinone moiety:** 110.5* (C-3'), 125.6 (C-5'), 126.1 (C-8'), 130.9 (C-8a'), 131.6 (C-4a'), 133.7 (C-7'), 134.6 (C-6'), 159.8* (C-2'), 179.6 (C-1'), 184.5 (C-4');<br>**HRMS (ESI):** *m/z* [M+Na]$^+$ calcd for $C_{16}H_{16}O_8Na$: 359.0737; found: 359.0734.
**5**	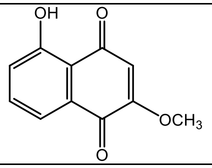	**5-hydroxy-2-methoxy-1,4-naphthoquinone (5)**<br>synthesized as described in Pelageev D.N., Dyshlovoy S.A., Pokhilo N.D., Denisenko V.A., Borisova K.L., Amsberg G.K., Bokemeyer C., Fedorov S.N., Honecker F., Anufriev V.Ph. Quinone-carbohydrate nonglucoside conjugates as a new type of cytotoxic agents: Synthesis and determination of in vitro activity // *Eur J Med Chem*, **2014**, *77*, 139-144.
**6**	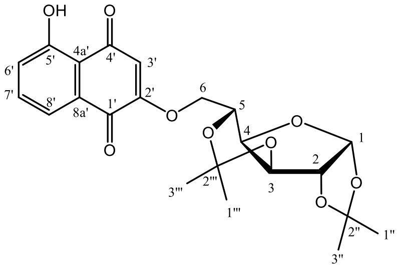	**1,2:3,5-di-O-isopropylidene-6-O-(5-hydroxy-1,4-naphthoquinone-2-yl)-*α*-D-glucofuranose (6):**<br>yellow solid;<br>yield 324 mg, 58% (recovery of unreacted 2,5-dihydroxy-1,4-naphthoquinone **5a** 134 mg);<br>mp 144-146°C;<br>**IR (CHCl$_3$) ν_{max}:** 2994, 2941, 1687, 1637, 1603, 1458, 1376, 1335, 1262, 1244 cm^{-1};<br>**^{1}H NMR (CDCl$_3$, 700 MHz) *δ*:** 1.34 (s, 3H, C(1'')H$_3$), 1.39 (s, 3H, C(1''')H$_3$), 1.40 (s, 3H, C(3''')H$_3$), 1.50 (s, 3H, C(3'')H$_3$), 3.98 (ddd, *J* = 7.8, 6.7, 2.3 Hz, 1H, H$_5$), 4.16 (dd, *J* = 11.0, 6.7 Hz, 1H, H$_{6a}$), 4.24 (dd, *J* = 11.0, 2.3 Hz, 1H, H$_{6b}$), 4.32 (d, *J* = 4.0 Hz, 1H, H$_3$), 4.49 (dd, *J* = 7.8, 4.0 Hz, 1H, H$_4$), 4.62 (d, *J* = 3.7 Hz, 1H, H$_2$), 6.02 (d, *J* = 3.7 Hz, 1H, H$_1$), 6.14 (s, 1H, H$_{3'}$), 7.26 (dd, *J* = 8.0, 0.9 Hz, 1H, H$_{6'}$), 7.57 (t, *J* = 8.0 Hz, 1H, H$_{7'}$), 7.64 (dd, *J* = 8.0, 0.9 Hz, 1H, H$_{8'}$), 12.19 (s, 3H, C(5')-OH);<br>**^{13}C NMR (176 MHz, CDCl$_3$) δ:** 23.7 (C-1'''), 24.0 (C-3'''), 26.5 (C-1''), 27.2 (C-3''), 69.6 (C-6), 70.3 (C-5), 75.1 (C-3), 78.8 (C-4), 84.0 (C-2), 101.4 (C-2'''), 106.5 (C-1), 110.3 (C-3'), 112.5 (C-2''), 114.1 (C-4a'), 119.4 (C-8'), 125.0 (C-6'), 131.1 (C-8a'), 135.4 (C-7'), 160.3 (C-2'), 161.0 (C-5'), 178.8 (C-1'), 190.8 (C-4');<br>**HRMS (EI):** *m/z* [M]$^+$ calcd for $C_{22}H_{24}O_9$: 432.1420; found: 432.1441.

**7**	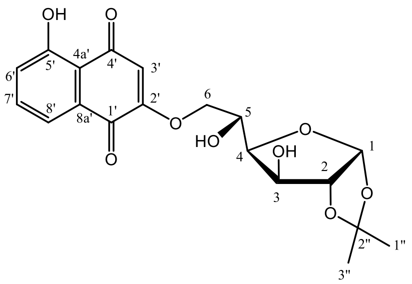	**1,2-O-isopropylidene-6-O-(5-hydroxy-1,4-naphthoquinone-2-yl)-*α*-D-glucofuranose (7):**<br>yellow solid;<br>yield 94 mg (96%);<br>mp 150-154°C;<br>**IR ($CHCl_3$) ν_{max}:** 3462, 2993, 2935, 1717, 1678, 1637, 1609, 1457, 1375, 1333, 1262, 1240 cm^{-1};<br>**^1H NMR ($CDCl_3$, 500 MHz) *δ*:** 1.35 (s, 3H, C(1'')H_3), 1.50 (s, 3H, C(3'')H_3), 3.89 (br d, *J* = 4.8 Hz, 1H, C(3)-OH), 4.04 (br s, 1H, C(5)-OH), 4.10 (dd, *J* = 10.0, 4.9 Hz, 1H, H_{6a}), 4.20 (dd, *J* = 6.3, 2.9 Hz, 1H, H_4), 4.34 (dd, *J* = 10.0, 2.6 Hz, 1H, H_{6b}), 4.48 (m, 1H, H_5), 4.51 (br m, 1H, H_3), 4.67 (d, *J* = 3.6 Hz, 1H, H_2), 6.05 (d, *J* = 3.6 Hz, 1H, H_1), 6.14 (s, 1H, $H_{3'}$), 7.31 (dd, *J* = 8.4, 1.0 Hz, 1H, $H_{6'}$), 7.59 (dd, *J* = 8.4, 7.5 Hz, 1H, $H_{7'}$), 7.66 (dd, *J* = 7.5, 1.0 Hz, 1H, $H_{8'}$), 12.18 (s, 1H, C(5')-OH);<br>**^{13}C NMR (125 MHz, $CDCl_3$) δ:** 26.2 (C-1''), 26.8 (C-3''), 68.4 (C-5), 71.5 (C-6), 76.3 (C-3), 79.6 (C-4), 85.4 (C-2), 104.9 (C-1), 110.3 (C-3'), 111.8 (C-2''), 114.1 (C-4a'), 119.7 (C-8'), 125.9 (C-6'), 130.7 (C-8a'), 135.6 (C-7'), 159.6 (C-2'), 161.3 (C-5'), 180.2 (C-1'), 190.3 (C-4');<br>**HRMS (EI):** *m/z* $[M]^+$ calcd for $C_{19}H_{20}O_9$: 392.1107; found: 392.1104.
**8**	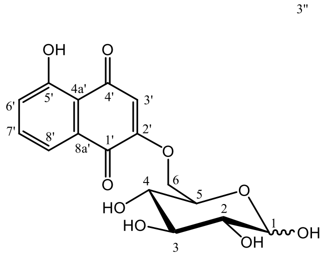	**6-O-(5-hydroxy-1,4-naphthoquinone-2-yl)-D-glucopyranose (8):**<br>yellow solid;<br>yield 31 mg (88%);<br>**^1H NMR (700 MHz, DMSO-d_6-D_2O, 10:1) δ:**<br>**carbohydrate moiety**<br>***α*-anomer:** 3.19 (dd, *J* = 9.3, 3.9 Hz, 1H, H_2), 3.22 (dd, *J* = 10.5, 9.3 Hz, 1H, H_4), 3.48 (t, *J* = 9.3 Hz, 1H, H_3), 3.94 (ddd, *J* = 10.5, 6.0, 1.7 Hz, 1H, H_5), 4.15 (dd, *J* = 11.2, 6.0 Hz, 1H, H_{6a}), 4.19 (dd, *J* = 11.2, 1.7 Hz, 1H, H_{6b}), 4.94 (d, *J* = 3.9 Hz, 1H, H_1);<br>**β-anomer:** 2.96 (dd, *J* = 8.7, 7.8 Hz, 1H, H_2), 3.21 (t, *J* = 8.7 Hz, 1H, H_3), 3.23 (t, *J* = 8.7 Hz, 1H, H_4), 3.52 (ddd, *J* = 8.7, 6.0, 1.0 Hz, 1H, H_5), 4.12 (dd, *J* = 11.4, 6.0 Hz, 1H, H_{6a}), 4.23 (dd, *J* = 11.4, 1.0 Hz, 1H, H_{6b}), 4.37 (d, *J* = 7.8 Hz, 1H, H_1);<br>**naphthoquinone moiety:** 6.30 (s, 1H, $H_{3'}$), 7.30 (d, *J* = 8.0 Hz, 1H, $H_{6'}$), 7.52 (d, *J* = 8.0 Hz, 1H, $H_{8'}$), 7.67 (t, *J* = 8.0 Hz, 1H, $H_{7'}$);<br>**^{13}C NMR (175 MHz, DMSO-d_6-D_2O, 10:1) δ:**<br>**carbohydrate moiety**<br>***α*-anomer:** 69.7 (C-5), 70.3 (C-6), 70.5 (C-4), 72.5 (C-2), 73.2 (C-3), 92.8 (C-1);<br>**β-anomer:** 70.1 (2C, C-4, C-6), 74.0 (C-5), 75.0 (C-2), 76.6 (C-3), 97.2 (C-1);<br>**naphthoquinone moiety:** 110.7* (C-3'), 114.3 (C-4a'), 119.6 (C-8'), 125.2 (C-6'), 131.6 (C-8a'), 136.6 (C-7'), 160.3 (C-5'), 161.1* (C-2'), 179.6 (C-1'), 191.4 (C-4');<br>**HRMS (ESI):** *m/z* $[M+Na]^+$ calcd for $C_{16}H_{16}O_9Na$: 375.0686; found: 375.0679.
**9**	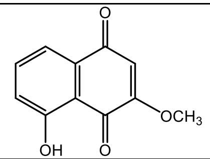	**8-hydroxy-2-methoxy-1,4-naphthoquinone (9)**<br>synthesized as described in Pelageev D.N., Dyshlovoy S.A., Pokhilo N.D., Denisenko V.A., Borisova K.L., Amsberg G.K., Bokemeyer C., Fedorov S.N., Honecker F., Anufriev V.Ph. Quinone-carbohydrate nonglucoside conjugates as a new type of cytotoxic agents: Synthesis and determination of in vitro activity // *Eur J Med Chem*, **2014**, *77*, 139-144.
**10**	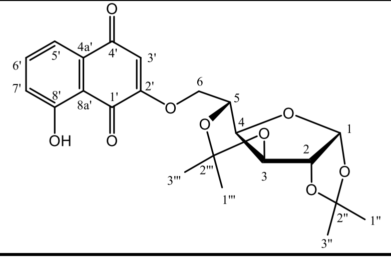	**1,2:3,5-di-O-isopropylidene-6-O-(8-hydroxy-1,4-naphthoquinone-2-yl)-*α*-D-glucofuranose (10):**<br>yellow solid;<br>yield 399 mg, 66% (recovery of unreacted 2,8-dihydroxy-1,4-naphthoquinone **9a** 114 mg);<br>mp 184-185°C;<br>**IR ($CHCl_3$) ν_{max}:** 2994, 2942, 1649, 1608, 1580, 1458, 1384, 1376, 1307, 1290, 1244 cm^{-1};<br>**^1H NMR ($CDCl_3$, 500 MHz) *δ*:** 1.34 (s, 3H, C(1'')H_3), 1.40 (s, 3H, C(1''')H_3), 1.41 (s, 3H, C(3''')H_3), 1.50 (s, 3H, C(3'')H_3), 3.99 (ddd, *J* = 7.7, 6.4, 2.5 Hz, 1H, H_5), 4.15 (dd, *J* = 11.0, 6.4 Hz, 1H, H_{6a}), 4.22 (dd, *J* = 11.0, 2.5 Hz, 1H, H_{6b}), 4.32 (d, *J* = 3.9 Hz, 1H, H_3), 4.49 (dd, *J* = 7.7, 3.9 Hz, 1H, H_4), 4.62 (d, *J* = 3.7 Hz, 1H, H_2), 6.03 (d, *J* = 3.7 Hz, 1H, H_1), 6.19 (s, 1H, $H_{3'}$), 7.23 (dd, *J* = 6.5, 3.0 Hz, 1H, $H_{7'}$), 7.61 (m, 2H,

		$H_{5'}$,$H_{6'}$), 11.77 (s, 1H, C(8')-OH);<br>**^{13}C NMR (125 MHz, CDCl$_3$) δ:** 23.7 (C-1'''), 24.0 (C-3'''), 26.5 (C-1''), 27.2 (C-3''), 69.5 (C-6), 70.3 (C-5), 75.1 (C-3), 78.9 (C-4), 84.1 (C-2), 101.4 (C-2'''), 106.5 (C-1), 111.3 (C-3'), 112.5 (C-2''), 114.3 (C-8a'), 118.8 (C-5'), 123.8 (C-7'), 132.0 (C-4a'), 137.0 (C-6'), 159.2 (C-2'), 161.8 (C-8'), 184.0 (C-4'), 184.5 (C-1');<br>**HRMS (EI):** *m/z* $[M]^+$ calcd for $C_{22}H_{24}O_9$: 432.1420; found: 432.1415.
11	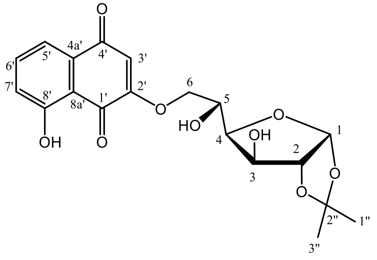	**1,2-O-isopropylidene-6-O-(8-hydroxy-1,4-naphthoquinone-2-yl)-α-D-glucofuranose (11):**<br>yellow solid;<br>yield 91 mg (93%);<br>mp 171-174°C;<br>**IR (CHCl$_3$) ν_{max}:** 3503, 2995, 2938, 1646, 1611, 1578, 1457, 1376, 1306, 1289, 1240 cm^{-1};<br>**^{1}H NMR (CDCl$_3$, 500 MHz) δ:** 1.33 (s, 3H, C(1'')H$_3$), 1.50 (s, 3H, C(3'')H$_3$), 3.69 (br d, *J* = 3.5 Hz, 1H, C(3)-O<u>H</u>), 3.98 (br s, 1H, C(5)-O<u>H</u>), 4.11 (dd, *J* = 10.0, 4.7 Hz, 1H, H_{6a}), 4.21 (dd, *J* = 6.3, 2.9 Hz, 1H, H_4), 4.33 (dd, *J* = 10.0, 2.6 Hz, 1H, H_{6b}), 4.48 (br s, 1H, H_5), 4.51 (br s, 1H, H_3), 4.63 (d, *J* = 3.7 Hz, 1H, H_2), 6.02 (d, *J* = 3.7 Hz, 1H, H_1), 6.18 (s, 1H, $H_{3'}$), 7.23 (dd, *J* = 7.8, 1.7 Hz, 1H, $H_{7'}$), 7.62 (dd, *J* = 7.8, 1.7 Hz, 1H, $H_{5'}$), 7.65 (t, *J* = 7.8, Hz, 1H, $H_{6'}$), 11.55 (s, 1H, C(8')-OH);<br>**^{13}C NMR (125 MHz, CDCl$_3$) δ:** 26.1 (C-1''), 26.8 (C-3''), 68.3 (C-5), 71.3 (C-6), 76.2 (C-3), 79.6 (C-4), 85.4 (C-2), 104.9 (C-1), 111.3 (C-3'), 111.9 (C-2''), 114.1 (C-8a'), 119.3 (C-5'), 123.9 (C-7'), 132.0 (C-4a'), 137.6 (C-6'), 158.7 (C-2'), 162.0 (C-8'), 183.5 (C-4'), 185.2 (C-1');<br>**HRMS (EI):** *m/z* $[M]^+$ calcd for $C_{19}H_{20}O_9$: 392.1107; found: 392.1111.
12	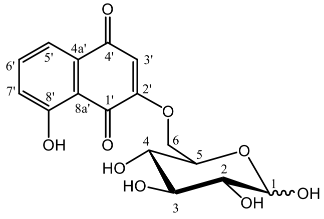	**6-O-(8-hydroxy-1,4-naphthoquinone-2-yl)-D-glucopyranose (12):**<br>yellow solid<br>yield 32 mg (91%);<br>**^{1}H NMR (500 MHz, DMSO-d$_6$) δ:**<br>**carbohydrate moiety**<br>**α-anomer:** 3.17 (dd, *J* = 9.0, 3.6 Hz, 1H, H_2), 3.22 (dd, *J* = 10.0, 9.0 Hz, 1H, H_4), 3.49 (t, *J* = 9.0 Hz, 1H, H_3), 3.97 (ddd, *J* = 10.0, 5.3, 1.8 Hz, 1H, H_5), 4.16 (dd, *J* = 10.5, 5.3 Hz, 1H, H_{6a}), 4.21 (dd, *J* = 10.5, 1.8 Hz, 1H, H_{6b}), 4.95 (d, *J* = 3.6 Hz, 1H, H_1);<br>**β-anomer:** 2.95 (dd, *J* = 8.8, 7.8 Hz, 1H, H_2), 3.20 (t, *J* = 8.8 Hz, 1H, H_3), 3.23 (t, *J* = 8.8 Hz, 1H, H_4), 3.53 (ddd, *J* = 8.8, 5.9, 1.7 Hz, 1H, H_5), 4.14 (dd, *J* = 11.0, 5.9 Hz, 1H, H_{6a}), 4.25 (dd, *J* = 11.0, 1.7 Hz, 1H, H_{6b}), 4.38 (d, *J* = 7.8 Hz, 1H, H_1);<br>**naphthoquinone moiety:** 6.36 (s, 1H, $H_{3'}$), 7.30 (dd, *J* = 7.4, 1.2 Hz, 1H, $H_{7'}$), 7.50 (dd, *J* = 7.4, 1.2 Hz, 1H, $H_{5'}$), 7.74 (t, *J* = 7.4 Hz, 1H, $H_{6'}$);<br>**^{13}C NMR (125 MHz, DMSO-d$_6$) δ:**<br>**carbohydrate moiety**<br>**α-anomer:** 69.2 (C-5), 69.9 (C-6), 70.2 (C-4), 72.2 (C-2), 72.9 (C-3), 92.5 (C-1);<br>**β-anomer:** 69.7 (C-6), 69.8 (C-4), 73.5 (C-5), 74.7 (C-2), 76.4 (C-3), 97.0 (C-1);<br>**naphthoquinone moiety:** 110.7* (C-3'), 114.5 (C-8a'), 118.0 (C-5'), 123.4 (C-7'), 132.0 (C-4a'), 137.1 (C-6'), 159.5* (C-2'), 160.6 (C-8'), 183.8 (C-4'), 184.1 (C-1');<br>**HRMS (ESI):** *m/z* $[M+Na]^+$ calcd for $C_{16}H_{16}O_9Na$: 375.0686; found: 375.0681.
13	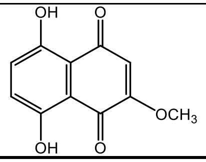	**5,8-dihydroxy-2-methoxy-1,4-naphthoquinone (13)**<br>synthesized as described in 1) Donaldson N. *The Chemistry and Technology of Naphthalene Compounds*. London (England): Arnold Ltd.; **1958**, 512; and 2) Yakubovskaya A.Y., Pokhilo N.D., Anufriev V.Ph., Anisimov M.M. Synthesis and antimicrobial and antifungal activities of compounds of the naphthazarin series // *Pharm Chem J*, **2009**, *43*, 396-398.

**14**	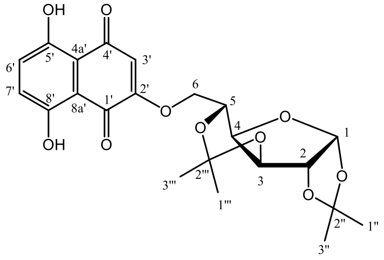	**1,2:3,5-di-O-isopropylidene-6-O-(5,8-dihydroxy-1,4-naphthoquinone-2-yl)-*α*-D-glucofuranose (14):**<br>red solid;<br>yield 170 mg, 24% (recovery of unreacted 2,5,8-trihydroxy-1,4-naphthoquinone **13a** 85 mg);<br>mp 155-159°C;<br>**IR (CHCl$_3$) ν_{max}:** 3489, 2994, 2937, 1608, 1572, 1456, 1385, 1376, 1309, 1293, 1241 cm^{-1};<br>**^{1}H NMR (CDCl$_3$, 500 MHz) *δ*:** 1.34 (s, 3H, C(1'')H_3), 1.40 (s, 3H, C(1''')H_3), 1.41 (s, 3H, C(3''')H_3), 1.51 (s, 3H, C(3'')H_3), 3.99 (ddd, *J* = 7.6, 6.7, 2.7 Hz, 1H, H_5), 4.17 (dd, *J* = 10.9, 6.7 Hz, 1H, H_{6a}), 4.25 (dd, *J* = 10.9, 2.7 Hz, 1H, H_{6b}), 4.32 (d, *J* = 4.0 Hz, 1H, H_3), 4.49 (dd, *J* = 7.6, 4.0 Hz, 1H, H_4), 4.62 (d, *J* = 3.7 Hz, 1H, H_2), 6.03 (d, *J* = 3.7 Hz, 1H, H_1), 6.21 (s, 1H, $H_{3'}$), 7.21 (d, *J* = 9.4 Hz, 1H, $H_{7'}$), 7.27 (d, *J* = 9.4 Hz, 1H, $H_{6'}$), 12.20 (s, 1H, C(8')-OH), 12.61 (s, 1H, C(5')-OH);<br>**^{13}C NMR (125 MHz, CDCl$_3$) δ:** 23.7 (C-1'''), 24.1 (C-3'''), 26.5 (C-1''), 27.2 (C-3''), 69.8 (C-6), 70.3 (C-5), 75.2 (C-3), 78.9 (C-4), 84.1 (C-2), 101.5 (C-2'''), 106.5 (C-1), 110.8 (C-4a'), 111.1 (C-3'), 111.6 (C-8a'), 112.5 (C-2''), 128.3 (C-7'), 130.6 (C-6'), 157.0 (C-5'), 158.5 (C-8'), 160.2 (C-2'), 182.1 (C-1'), 188.2 (C-4');<br>**HRMS (EI):** *m/z* [M]$^+$ calcd for $C_{22}H_{24}O_{10}$: 448.1369; found: 448.1365.
**15**	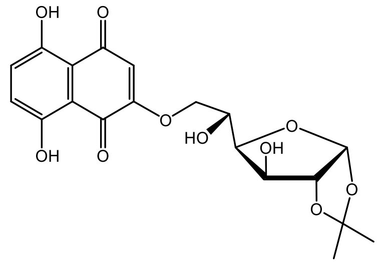	**1,2-O-isopropylidene-6-O-(5,8-dihydroxy-1,4-naphthoquinone-2-yl)-*α*-D-glucofuranose (15)**<br>red solid;<br>yield 89 mg (87%);<br>mp 128-131°C;<br>**IR (CHCl$_3$) ν_{max}:** 3488, 3058, 3004, 2931, 1607, 1456, 1455 cm^{-1};<br>**^{1}H NMR (CDCl$_3$, 500 MHz) *δ*:** 1.33 (s, 3H, C(1'')H_3), 1.50 (s, 3H, C(3'')H_3), 3.69 (br d, *J* = 3.5 Hz, 1H, C(3)-O<u>H</u>), 3.98 (br s, 1H, C(5)-O<u>H</u>), 4.11 (dd, *J* = 10.0, 4.7 Hz, 1H, H_{6a}), 4.21 (dd, *J* = 6.3, 2.9 Hz, 1H, H_4), 4.33 (dd, *J* = 10.0, 2.6 Hz, 1H, H_{6b}), 4.48 (br s, 1H, H_5), 4.51 (br s, 1H, H_3), 4.63 (d, *J* = 3.7 Hz, 1H, H_2), 6.02 (d, *J* = 3.7 Hz, 1H, H_1), 6.21 (s, 1H, $H_{3'}$), 7.22 (d, *J* = 9.4 Hz, 1H, $H_{7'}$), 7.31 (d, *J* = 9.4 Hz, 1H, $H_{6'}$), 12.03 (s, 1H, C(8')-OH), 12.60 (s, 1H, C(5')-OH);<br>**^{13}C NMR (125 MHz, CDCl$_3$) δ:** 26.1 (C-1''), 26.8 (C-3''), 68.3 (C-5), 71.3 (C-6), 76.2 (C-3), 79.6 (C-4), 85.4 (C-2), 104.9 (C-1), 110.8 (C-4a'), 111.1 (C-3′), 111.4 (C-8a′), 111.9 (C-2''), 128.4 (C-7'), 131.1 (C-6'), 159.7 (C-2'), 157.4 (C-5'), 158.7 (C-8'), 182.8 (C-1'), 187.7 (C-4');<br>**HRMS (EI):** *m/z* [M]$^+$ calcd for $C_{19}H_{20}O_{10}$: 408.1056; found: 408.1053.
**16**	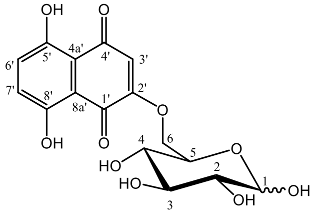	**6-O-(5,8-hydroxy-1,4-naphthoquinone-2-yl)-D-glucopyranose (16):**<br>red solid<br>yield 27 mg (73%);<br>**^{1}H NMR (500 MHz, DMSO-d$_6$-D$_2$O, 10:1) *δ*:**<br>**carbohydrate moiety**<br>***α*-anomer:** 3.18 (dd, *J* = 9.1, 3.6 Hz, 1H, H_2), 3.22 (dd, *J* = 9.6, 9.1 Hz, 1H, H_4), 3.48 (t, *J* = 9.1 Hz, 1H, H_3), 3.95 (ddd, *J* = 9.6, 4.5, 1.4 Hz, 1H, H_5), 4.18 (dd, *J* = 11.0, 4.5 Hz, 1H, H_{6a}), 4.22 (dd, *J* = 11.0, 1.4 Hz, 1H, H_{6b}), 4.94 (d, *J* = 3.6 Hz, 1H, H_1);<br>**β-anomer:** 2.95 (dd, *J* = 8.6, 7.8 Hz, 1H, H_2), 3.21 (t, *J* = 8.6 Hz, 1H, H_3), 3.23 (t, *J* = 8.6 Hz, 1H, H_4), 3.53 (ddd, *J* = 8.6, 5.7, 1.4 Hz, 1H, H_5), 4.16 (dd, *J* = 11.0, 5.7 Hz, 1H, H_{6a}), 4.26 (dd, *J* = 11.0, 1.4 Hz, 1H, H_{6b}), 4.38 (d, *J* = 7.8 Hz, 1H, H_1);<br>**naphthoquinone moiety:** 6.40 (s, 1H, $H_{3'}$), 7.32 (d, *J* = 9.3 Hz, 1H, $H_{7'}$), 7.37 (d, *J* = 9.3 Hz, 1H, $H_{6'}$);<br>**^{13}C NMR (125 MHz, DMSO-d$_6$-D$_2$O, 10:1) δ:**<br>**carbohydrate moiety**<br>***α*-anomer:** 69.5 (C-5), 70.3 (2C, C-4, C-6), 72.3 (C-2), 73.0 (C-3), 92.6 (C-1);<br>**β-anomer:** 69.6 (C-4), 70.1 (C-6), 73.8 (C-5), 74.8 (C-2), 76.5 (C-3), 97.1 (C-1);<br>**naphthoquinone moiety:** 111.1* (2C, C-3', C-4a'), 112.0 (C-8a'), 128.4 (C-7'), 130.4 (C-6'), 155.6 (C-5'), 156.9 (C-8'), 160.7* (C-2'), 182.5 (C-1'), 189.1 (C-4');<br>**HRMS (ESI):** *m/z* [M+Na]$^+$ calcd for $C_{16}H_{16}O_{10}Na$: 391.0636; found: 391.0631.

**17**	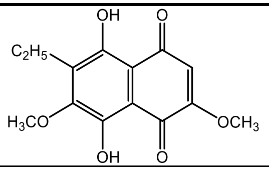	**6-ethyl-5,8-dihydroxy-2,7-dimethoxy-1,4-naphthoquinone (17)**<br>synthesized as described in Glazunov V.P., Tchizhova A.Ya., Pokhilo N.D., Anufriev V.Ph., Elyakov G.B. First direct observation of tautomerism of monohydroxynaphthazarins by IR-spectroscopy // *Tetrahedron*, **2002**, *58*, 1751-1757.
**18**	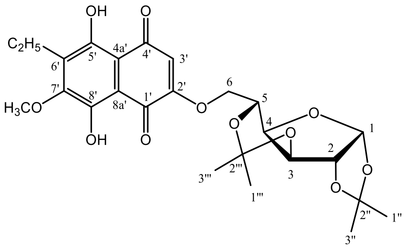	**1,2:3,5-di-O-isopropylidene-6-O-(6-ethyl-5,8-dihydroxy-7-methoxy-1,4-naphthoquinone-2-yl)-α-D-glucofuranose (18):**<br>red solid;<br>yield 346 mg, 54% (recovery of unreacted 2,5,8-trihydroxy-1,4-naphthoquinone **17a** 193 mg);<br>mp 113-115°C;<br>**IR (CHCl$_3$) ν_{max}:** 3055, 2994, 2941, 1606, 1454, 1425, 1405, 1385, 1377, 1282, 1163 cm^{-1};<br>**^{1}H NMR (CDCl$_3$, 700 MHz) δ:** 1.16 (t, *J* = 7.5 Hz, 3H, C<u>H$_3$</u>CH$_2$), 1.34 (s, 3H, C(1'')H$_3$), 1.40 (s, 3H, C(1''')H$_3$), 1.41 (s, 3H, C(3''')H$_3$), 1.51 (s, 3H, C(3'')H$_3$), 2.71 (q, *J* = 7.5 Hz, 2H, CH$_3$C<u>H$_2$</u>), 3.99 (ddd, *J* = 7.7, 6.7, 2.4 Hz, 1H, H$_5$), 4.05 (s, 3H, OCH$_3$), 4.18 (dd, *J* = 10.8, 6.7 Hz, 1H, H$_{6a}$), 4.26 (dd, *J* = 10.8, 2.4 Hz, 1H, H$_{6b}$), 4.32 (d, *J* = 4.0 Hz, 1H, H$_3$), 4.49 (dd, *J* = 7.7, 4.0 Hz, 1H, H$_4$), 4.62 (d, *J* = 3.7 Hz, 1H, H$_2$), 6.03 (d, *J* = 3.7 Hz, 1H, H$_1$), 6.31 (s, 1H, H$_{3'}$), 12.78 (s, 1H, C(8')-OH), 13.27 (s, 1H, C(5')-OH);<br>**^{13}C NMR (176 MHz, CDCl$_3$) δ:** 13.5 (<u>C</u>H$_3$CH$_2$), 17.3 (CH$_3$<u>C</u>H$_2$), 23.8 (C-1'''), 24.1 (C-3'''), 26.5 (C-1''), 27.2 (C-3''), 61.4 (O<u>C</u>H$_3$), 69.7 (C-6), 70.4 (C-5), 75.2 (C-3), 78.9 (C-4), 84.1 (C-2), 101.4 (C-2'''), 106.1 (C-4a'), 106.5 (C-1), 110.3 (C-3'), 111.0 (C-8a'), 112.4 (C-2''), 138.6 (C-6'), 154.9 (C-7'), 158.8 (C-2'), 163.6 (C-8'), 167.0 (C-5'), 172.2 (C-1'), 178.5 (C-4');<br>**HRMS (EI):** *m/z* [M]$^+$ calcd for $C_{25}H_{30}O_{11}$: 506.1788; found: 506.1795.
**19**	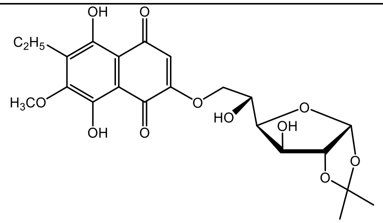	**1,2-O-isopropylidene-6-O-(6-ethyl-5,8-dihydroxy-7-methoxy-1,4-naphthoquinone-2-yl)-α-D-glucofuranose (19)**<br>spectral data of the synthesized compound was identical to the ones described in Pokhilo N.D., Atopkina L.N., Kiseleva M.I., Denisenko V.A., Anufriev V.Ph. Synthesis and Cytotoxic Evaluation of Glucoconjugated Ethylmompain Derivatives // *Nat Prod Commun*, **2017**, *12*, 1475–1478.
**20**	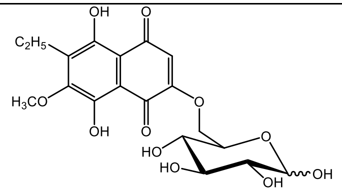	**6-O-(6-ethyl-5,8-dihydroxy-7-methoxy-1,4-naphthoquinone-2-yl)-D-glucopyranose (20)**<br>spectral data of the synthesized compound was identical to the ones described in Pokhilo N.D., Atopkina L.N., Kiseleva M.I., Denisenko V.A., Anufriev V.Ph. Synthesis and Cytotoxic Evaluation of Glucoconjugated Ethylmompain Derivatives // *Nat Prod Commun*, **2017**, *12*, 1475–1478.
**21**	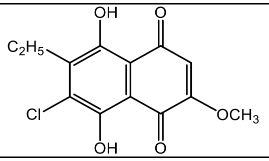	**7-chloro-6-ethyl-5,8-dihydroxy-2-methoxy-1,4-naphthoquinone (21)**<br>synthesized as described in Pokhilo N.D., Atopkina L.N., Kiseleva M.I., Denisenko V.A., Anufriev V.Ph. Synthesis and Cytotoxic Evaluation of Glucoconjugated Ethylmompain Derivatives // *Nat Prod Commun*, **2017**, *12*, 1475–1478.
**22**		**1,2:3,5-di-O-isopropylidene-6-O-(7-chloro-6-ethyl-5,8-dihydroxy-1,4-naphthoquinone-2-yl)-α-D-glucofuranose (22):**<br>red solid;<br>yield 400 mg, 57% (recovery of unreacted 7-chloro-6-ethyl-2,5,8-trihydroxy-1,4-naphthoquinone **21a** 167 mg);

	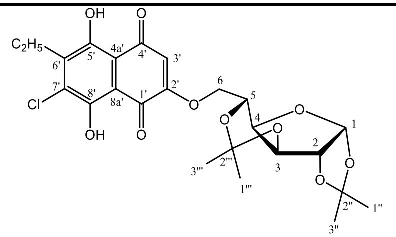	mp 95-98°C;<br>**IR ($CHCl_3$) ν_{max}:** 2993, 2940, 1610, 1449, 1420, 1399, 1385, 1377, 1305, 1268, 1246 cm^{-1};<br>**^{1}H NMR ($CDCl_3$, 700 MHz) δ:** 1.21 (t, J = 7.5 Hz, 3H, C<u>H</u>$_3$CH$_2$), 1.34 (s, 3H, C(1'')H$_3$), 1.40 (s, 3H, C(1''')H$_3$), 1.41 (s, 3H, C(3''')H$_3$), 1.51 (s, 3H, C(3'')H$_3$), 2.94 (q, J = 7.5 Hz, 2H, CH$_3$C<u>H$_2$</u>), 3.99 (ddd, J = 7.7, 6.6, 2.4 Hz, 1H, H$_5$), 4.19 (dd, J = 10.8, 6.6 Hz, 1H, H$_{6a}$), 4.27 (dd, J = 10.8, 2.4 Hz, 1H, H$_{6b}$), 4.32 (d, J = 4.0 Hz, 1H, H$_3$), 4.49 (dd, J = 7.7, 4.0 Hz, 1H, H$_4$), 4.63 (d, J = 3.7 Hz, 1H, H$_2$), 6.03 (d, J = 3.7 Hz, 1H, H$_1$), 6.28 (s, 1H, H$_{3'}$), 12.87 (s, 1H, C(8')-OH), 13.18 (s, 1H, C(5')-OH);<br>**^{13}C NMR (176 MHz, $CDCl_3$) δ:** 12.1 (<u>C</u>H$_3$CH$_2$), 21.4 (CH$_3$<u>C</u>H$_2$), 23.7 (C-1'''), 24.0 (C-3'''), 26.5 (C-1''), 27.2 (C-3''), 69.9 (C-6), 70.3 (C-5), 75.2 (C-3), 78.9 (C-4), 84.1 (C-2), 101.5 (C-2'''), 106.5 (C-1), 108.0 (C-4a'), 110.2 (C-8a'), 110.6 (C-3'), 112.5 (C-2''), 133.3 (C-7'), 146.4 (C-6'), 159.4 (2C, C-2', C-8'), 160.6 (C-5'), 176.4 (C-1'), 183.2 (C-4');<br>**HRMS (EI):** m/z [M]$^+$ calcd for $C_{24}H_{27}O_{10}Cl$: 510.1293; found: 510.1289.
**23**	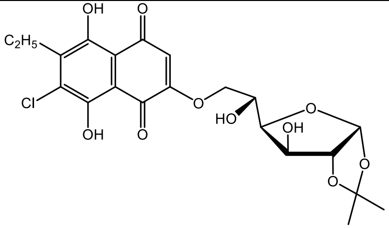	**1,2-O-isopropylidene-6-O-(7-chloro-6-ethyl-5,8-dihydroxy-1,4-naphthoquinone-2-yl)-α-D-glucofuranose (23)**<br>spectral data of the synthesized compound was identical to the ones described in Pokhilo N.D., Atopkina L.N., Kiseleva M.I., Denisenko V.A., Anufriev V.Ph. Synthesis and Cytotoxic Evaluation of Glucoconjugated Ethylmompain Derivatives // *Nat Prod Commun*, **2017**, *12*, 1475−1478.
**24**	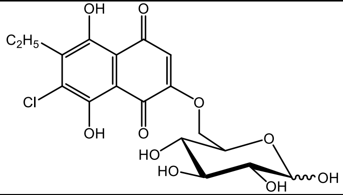	**6-O-(7-chloro-6-ethyl-5,8-dihydroxy-1,4-naphthoquinone-2-yl)-D-glucopyranose (24)**<br>spectral data of the synthesized compound was identical to the ones described in Pokhilo N.D., Atopkina L.N., Kiseleva M.I., Denisenko V.A., Anufriev V.Ph. Synthesis and Cytotoxic Evaluation of Glucoconjugated Ethylmompain Derivatives // *Nat Prod Commun*, **2017**, *12*, 1475−1478.
**25**	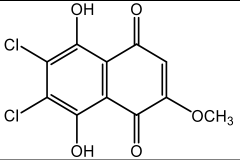	**6,7-dichloro-5,8-dihydroxy-2-methoxy-1,4-naphthoquinone (25)**<br>synthesized as described in Glazunov V.P., Tchizhova A.Ya., Shuvalova M.I., Anufriev V.Ph. Chemistry of naphthazarin derivatives. 7. Determination of structures of substituted 2,6(7)-dihydroxynaphthazarins by UV and IR spectroscopy // *Rus Chem Bull*, **2001**, *50*, 88-94.
**26**	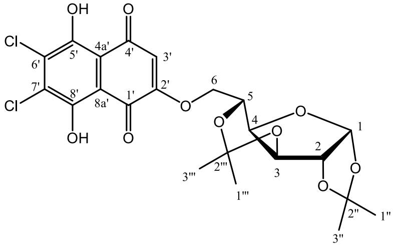	**1,2:3,5-di-O-isopropylidene-6-O-(6,7-dichloro-5,8-dihydroxy-1,4-naphthoquinone-2-yl)-α-D-glucofuranose (26):**<br>red solid;<br>yield 133 mg, 14% (recovery of unreacted 6,7-dichloro-2,5,8-trihydroxy-1,4-naphthoquinone **25a** 43 mg);<br>mp 173-176°C;<br>**IR ($CHCl_3$) ν_{max}:** 2993, 2941, 1617, 1601, 1559, 1472, 1405, 1385, 1351, 1301, 1272, 1241 cm^{-1};<br>**^{1}H NMR ($CDCl_3$, 300 MHz) δ:** 1.34 (s, 3H, C(1'')H$_3$), 1.40 (s, 3H, C(1''')H$_3$), 1.41 (s, 3H, C(3''')H$_3$), 1.51 (s, 3H, C(3'')H$_3$), 3.99 (ddd, J = 7.7, 6.5, 2.6 Hz, 1H, H$_5$), 4.20 (dd, J = 10.8, 6.5 Hz, 1H, H$_{6a}$), 4.29 (dd, J = 10.8, 2.6 Hz, 1H, H$_{6b}$), 4.32 (d, J = 4.0 Hz, 1H, H$_3$), 4.49 (dd, J = 7.7, 4.0 Hz, 1H, H$_4$), 4.63 (d, J = 3.7 Hz, 1H, H$_2$), 6.03 (d, J = 3.7 Hz, 1H, H$_1$), 6.32 (s, 1H, H$_{3'}$), 12.76 (s, 1H, C(8')-OH), 13.23 (s, 1H, C(5')-OH);<br>**^{13}C NMR (75 MHz, $CDCl_3$) δ:** 23.7 (C-1'''), 24.0 (C-3'''), 26.5 (C-1''), 27.2 (C-3''), 70.1 (C-6), 70.3 (C-5), 75.2 (C-3), 78.9 (C-4), 84.0 (C-2), 101.5 (C-2'''), 106.5 (C-1), 108.4 (C-4a'), 110.1 (C-8a'), 110.4 (C-3'), 112.5 (C-2''), 133.3 (C-7'), 135.6 (C-6'), 157.0 (C-5'), 158.3 (C-8'), 159.8 (C-2'), 177.4 (C-1'), 183.8 (C-4');<br>**HRMS (EI):** m/z [M]$^+$ calcd for $C_{22}H_{22}O_{10}Cl_2$: 516.0590; found: 516.0584.

**27**	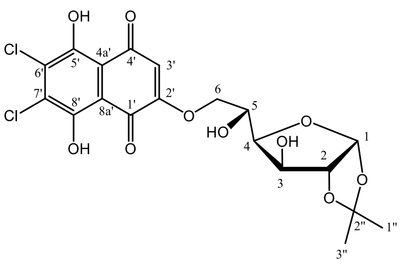	**1,2-O-isopropylidene-6-O-(6,7-dichloro-5,8-dihydroxy-1,4-naphthoquinone-2-yl)-α-D-glucofuranose (27):**<br>red solid;<br>yield 89 mg (75%);<br>mp 178-180°C;<br>**IR ($CHCl_3$) ν_{max}:** 3504, 2996, 2937, 1615, 1604, 1557, 1471, 1377, 1300, 1271, 1239 cm^{-1};<br>**^{1}H NMR ($CDCl_3$, 500 MHz) δ:** 1.33 (s, 3H, C(1'')H_3), 1.50 (s, 3H, C(3'')H_3), 3.69 (br d, *J* = 3.5 Hz, 1H, C(3)-O<u>H</u>), 3.98 (br s, 1H, C(5)-O<u>H</u>), 4.11 (dd, *J* = 10.0, 4.7 Hz, 1H, H_{6a}), 4.21 (dd, *J* = 6.3, 2.9 Hz, 1H, H_4), 4.33 (dd, *J* = 10.0, 2.6 Hz, 1H, H_{6b}), 4.48 (br s, 1H, H_5), 4.51 (br s, 1H, H_3), 4.62 (d, *J* = 3.7 Hz, 1H, H_2), 6.02 (d, *J* = 3.7 Hz, 1H, H_1), 6.34 (s, 1H, $H_{3'}$), 12.65 (s, 1H, C(8')-OH), 13.22 (s, 1H, C(5')-OH);<br>**^{13}C NMR (125 MHz, $CDCl_3$) δ:** 26.2 (C-1''), 26.8 (C-3''), 67.8 (C-5), 71.9 (C-6), 75.6 (C-3), 79.8 (C-4), 85.2 (C-2), 105.1 (C-1), 108.4 (C-4a'), 109.9 (C-8a'), 110.4 (C-3'), 111.9 (C-2''), 133.4 (C-7'), 136.1 (C-6'), 157.4 (C-5'), 158.5 (C-8'), 159.2 (C-2'), 178.1 (C-1'), 183.3 (C-4');<br>**HRMS (EI):** *m/z* [M]$^+$ calcd for $C_{19}H_{18}Cl_2O_{10}$: 476.0277; found: 476.0271.
**28**	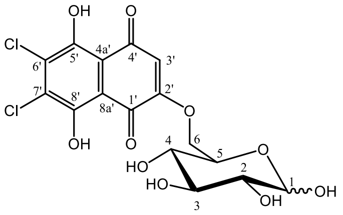	**6-O-(6,7-dichloro-5,8-dihydroxy-1,4-naphthoquinone-2-yl)-D-glucopyranose (28):**<br>red solid<br>yield 35 mg (80%);<br>**^{1}H NMR (500 MHz, DMSO-d_6) δ:**<br>**carbohydrate moiety**<br>**α-anomer:** 3.18 (dd, *J* = 9.0, 3.6 Hz, 1H, H_2), 3.22 (dd, *J* = 10.0, 9.0 Hz, 1H, H_4), 3.49 (t, *J* = 9.0 Hz, 1H, H_3), 3.98 (ddd, *J* = 10.0, 4.8, 2.3 Hz, 1H, H_5), 4.25 (dd, *J* = 10.5, 4.8 Hz, 1H, H_{6a}), 4.29 (dd, *J* = 10.5, 2.3 Hz, 1H, H_{6b}), 4.95 (d, *J* = 3.6 Hz, 1H, H_1);<br>**β-anomer:** 2.96 (dd, *J* = 8.8, 7.8 Hz, 1H, H_2), 3.20 (t, *J* = 8.8 Hz, 1H, H_3), 3.23 (t, *J* = 8.8 Hz, 1H, H_4), 3.54 (ddd, *J* = 8.8, 5.9, 1.7 Hz, 1H, H_5), 4.24 (dd, *J* = 11.0, 5.9 Hz, 1H, H_{6a}), 4.32 (dd, *J* = 11.0, 1.7 Hz, 1H, H_{6b}), 4.38 (d, *J* = 7.8 Hz, 1H, H_1);<br>**naphthoquinone moiety:** 6.60 (s, 1H, $H_{3'}$), 12.38 (s, 1H, C(8')-OH), 13.17 (s, 1H, C(5')-OH);<br>**^{13}C NMR (125 MHz, DMSO-d_6-D_2O, 10:1) δ:**<br>**carbohydrate moiety**<br>**α-anomer:** 69.3 (C-5), 69.8 (C-4), 70.5 (C-6), 72.2 (C-2), 72.9 (C-3), 92.5 (C-1);<br>**β-anomer:** 70.2 (C-4), 70.3 (C-6), 73.5 (C-5), 74.7 (C-2), 76.5 (C-3), 97.0 (C-1);<br>**naphthoquinone moiety:** 109.2* (C-4a'), 110.1* (C-3'), 111.1 (C-8a'), 131.3* (C-7'), 133.0* (C-6'), 155.5* (C-5'), 156.4* (C-8'), 160.1* (C-2'), 177.1* (C-1'), 184.3* (C-4');<br>**HRMS (ESI):** *m/z* [M+Na]$^+$ calcd for $C_{16}H_{14}Cl_2O_{10}Na$: 458.9856; found: 458.9844.

* The signals of these carbon atoms of naphthoquinone moieties in deprotected derivatives are doubled and cannot be uniquely assigned.

Biology

Reagents and Antibodies

Anisomycin was purchased from NeoCorp (Weilheim, Germany). z-VAD(OMe)-fmk was purchased from Enzo Life Sciences (Farmingdale, NY, USA). Annexin-V-FITC was purchased from BD Bioscience (San Jose, CA, USA). MTT (3-(4,5-dimethylthiazol-2-yl)-2,5-diphenyltetrazolium bromide) and propidium iodide (PI) were purchased from Sigma (Taufkirchen, Germany). H_2O_2 was purchased from Carl Roth (Karlsruhe, Germany). cOmplete™ *EASY*packs protease inhibitors cocktail and PhosSTOP™ *EASY*packs were purchased from Roche (Mannheim, Germany). CCCP (2-[2-(3-chlorophenyl)hydrazinylyidene]propanedinitrile) was purchased from Sigma (Taufkirchen, Germany). JC-1 (5,5',6,6'-Tetrachloro-1,1',3,3'-tetraethylbenzimidazolylcarbocyanine iodide) was purchased from AdiloGen Life Science (Epalinges, Switzerland).

Primary and secondary antibodies used are listed in Table S3.

Table S3. List of antibodies used.

Antibodies	Clonality	Source	Cat.-No.	Dilution	Manufacturer
anti-ERK1/2	mAb	mouse	#9107	1:2000	Cell Signaling
anti-JNK1/2	mAb	rabbit	#9258	1:1000	Cell Signaling
anti-p38	mAb	rabbit	#9212	1:1000	Cell Signaling
anti-phospho-ERK1/2	mAb	rabbit	#4377	1:1000	Cell Signaling
anti-phospho-JNK1/2	mAb	rabbit	#4668	1:1000	Cell Signaling
anti-phospho-p38	mAb	rabbit	#4511	1:1000	Cell Signaling
anti-phospho-Akt	mAb	rabbit	#4058	1:1000	Cell Signaling
anti-Akt	pAb	rabbit	#9272	1:1000	Cell Signaling
anti-phospho-MEK1/2	mAb	rabbit	#2338	1:1000	Cell Signaling
anti-MEK1/2	pAb	rabbit	#9122	1:1000	Cell Signaling
anti-α-Tubulin	mAb	mouse	T5168	1:5000	Sigma-Aldrich
anti-β-Actin-HRP	pAb	goat	sc-1616	1:10000	Santa Cruz
anti-AIF	mAb	rabbit	#5318	1:1000	Cell Signaling
anti-AR	pAb	rabbit	sc-816	1:200	Santa Cruz
anti-AR-V7	mAb	rabbit	198394	1:1000	abcam
anti-Bax	mAb	rabbit	#5023	1:1000	Cell Signaling
anti-Bcl-2	pAb	rabbit	#2876	1:1000	Cell Signaling
anti-cleaved Caspase-3	mAb	rabbit	#9664	1:1000	Cell Signaling
anti-cleaved Caspase-9	mAb	rabbit	#20750	1:1000	Cell Signaling
anti-LC3B-I/II	pAb	rabbit	#2775	1:1000	Cell Signaling
anti-cytochrome C	mAb	rabbit	#11940	1:1000	Cell Signaling
anti-p21$^{Waf1/Cip1}$	mAb	rabbit	#2947	1:1000	Cell Signaling
anti-PARP	pAb	rabbit	#9542	1:1000	Cell Signaling
anti-SQSTM/p62	pAb	rabbit	#5114	1:1000	Cell Signaling
anti-Survivin	pAb	rabbit	NB500-201	1:1000	Novus
anti-mouse IgG-HRP		sheep	NXA931	1:10000	GE Healthcare
anti-rabbit IgG-HRP		goat	#7074	1:5000	Cell Signaling

Cell Culture Conditions

Cells were incubated at 37°C in a humidified atmosphere with 5% (v/v) CO_2. Cells were continuously kept in culture for a maximum of 3 months, and were routinely inspected microscopically for stable phenotype and regularly checked for contamination with mycoplasma.

PC-3, DU145, LNCaP, 22Rv1 and PNT2 cells were cultured in 10% FBS/RPMI medium (RPMI medium supplemented with Glutamax™-I (Invitrogen, Paisley, UK) containing 10% fetal bovine

serum (FBS, Invitrogen) and 1% penicillin/streptomycin (Invitrogen)). MRC-9, HEK 293 and VCaP cells were cultured in 10% FBS/DMEM medium (DMEM medium supplemented with Glutamax™-I (Invitrogen) containing 10% FBS and 1% penicillin/streptomycin (Invitrogen)). RWPE-1 cells were cultured in Clonetics® EGM™-2 SingleQuots® medium (Lonza, Walkersville, MD, USA) containing 10% FBS. HUVEC cells (passage 11) were cultured in Clonetics® EGM™-2 SingleQuots® medium (Lonza, Walkersville, MD, USA) containing 10% FBS.

Methods of Proteomics

LC-MS Sample Preparation

Cell lines were lysed in 100 mM triethylammonium bicarbonate and 1% *w*/*v* sodium deoxycholate buffer followed by probe sonication to destroy polynucleotides and heat induce protein denaturing at 99 °C for 5 min. Protein concentrations were estimated with a bicinchonic acid (BCA) protein assay (Thermo Fisher Scientific, Bremen, Germany). 20 µg of protein were reduced in presence of 10 mM dithiotreithol (Sigma Aldrich) for 30 min at 60 °C followed by cysteine alkylation with 20 mM iodo acetamide (Sigma Aldrich) for 30 min at 37 °C in the dark and enzymatic degradation with sequencing grade trypsin (Promega) over night at 37 °C. Digestion was quenched with 1% formic acid (FA), precipitated sodium deoxycholate removed by centrifugation for 5 min at 14,000 g and the supernatant was dried in a vacuum centrifuge.

LC-MS/MS in Data Dependent and Data Independent Mode

Samples were resuspended in 0.1% FA and transferred into a full recovery autosampler vial (Waters). Chromatographic separation was achieved on a nano-UPLC system (Acquity, Waters Corporation, Milford, MS, USA) with a two-buffer-system (buffer A: 0.1% FA in water, buffer B: 0.1% FA in Acetonitrile (ACN), both at pH = 3). Attached to the UPLC was a reversed-phase peptide trapping column (Symmetry C18, 180 µm × 20 mm, 100 Å pore size, 5 µm particle size) for desalting, followed by a reversed-phase capillary separation of the tryptic peptides (BEH C18; 75 µm × 200 mm, 130 Å pore size, 1.7 µm particle size), using a 60 min gradient with increasing ACN concentration from 2–30% ACN. The eluting peptides were analyzed on a quadrupole-orbitrap mass spectrometer (QExactive, Thermo Fisher Scientific, Bremen, Germany) in data dependent acquisition (DDA) and data independent acquisition (DIA) for quantification.

In DDA mode, one replicate of each cell line was randomly chosen and 1 µg per sample was used per LC-MS/MS run to build a reference spectral library used for data extraction of samples acquired in DIA mode. For DDA, the 12 most intense ions per precursor scan (1×10^6 ions, 70,000 Resolution, 120 ms fill time) were analyzed by MS/MS (HCD at 25 normalized collision energy, 1×10^5 ions, 17,500 Resolution, 50 ms fill time) in a range of 400–1300 m/z. A dynamic precursor exclusion of 20 s was used. For DIA, 1 µg of each sample was analyzed using a 30 sequential 20 Da fixed window method covering the mass range from 400–1000 m/z. Per cycle, 2 precursor scans (1×10^6 ions, 35,000 Resolution, 110 ms fill time, m/z range 390–1010 m/z) and 30 MS/MS scans (HCD at 28 normalized collision energy, 1×10^6 ions, 17,500 Resolution, 50 ms fill time) were performed. After the first precursor scan, 15 MS/MS scans were performed covering the precursor mass range from 400–700 m/z followed by the second precursor scan and another 15 MS/MS scans ranging from 700–1000 m/z.

LC-MS/MS Data Processing and Analysis

Acquired DDA LC-MS/MS data were searched against the human SwissProt protein data base downloaded from Uniprot protein database (release December 2018, EMBL, Hinxton, Great Britain) using the search engine Sequest integrated in the protein identification software "Proteome Discoverer" (version 2.0, Thermo Fisher Scientific, Bremen, Germany). Mass tolerances for precursors was set to 10 ppm and 0.02 Da for fragments. Carbamidomethylation was set as a fixed modification for cysteine residues and the oxidation of methionine, pyro-glutamate formation at glutamine residues at the peptide N-terminus as well as acetylation of the protein N-terminus,

methionine loss at the protein N-terminus and the Acetylation after methionine loss at the protein N-terminus were allowed as variable modifications. Only peptide with a high confidence (false discovery rate < 1% using a decoy data base approach) were accepted as identified.

Proteome Discoverer search results were imported into Skyline quantification software for processing of DIA LC-MS data (version 4.2, MacCoss Lab Software, University of Washington, USA) allowing only high confidence peptides with more than 4 fragment ions. A maximum of 5 fragment ions per peptide were used for information extraction from DIA files for peptides with a dot product of > 0.85. Peptide peak areas were summed to generate protein areas which were then used for relative abundance comparison. Protein areas were imported into a Perseus statistical analysis software (version 1.5.8; Max Planck Institute of Biochemistry; Munich, Germany) (Tyanova et al. Nature Methods. 2016. 13(9):731-40).

Table S4. Cytotoxic activity of the synthesized compounds. Cytotoxicity was determined in human prostate cancer PC-3 cells versus human prostate non-cancer PNT2 cells after 48 h of treatment, as well as its ratio. IC_{50} was evaluated using MTT assay. SI–selectivity index. #–the IC_{50}s in both cell lines were >100 μM. Anisomycin was used as a reference substance.

No	Formula	IC_{50}(PC-3)	IC_{50}(PNT2)	SI<br>[IC_{50}(PNT2) / IC_{50}(PC-3)]
**1**	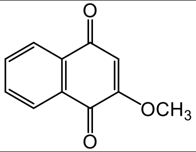	9.33	14.47	1.55
**2**	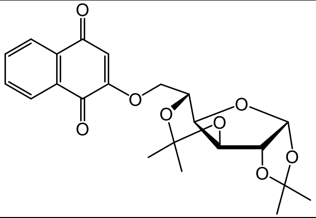	13.12	16.23	1.24
**3**	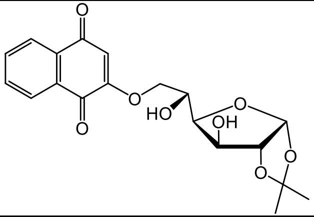	24.25	31.1	1.28
**4**	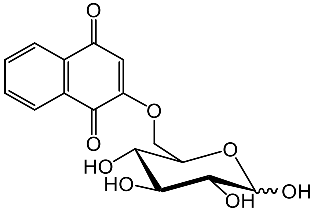	>100	>100	#
**5**	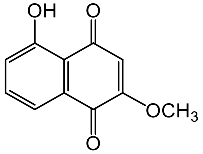	4.46	5.84	1.31

**6**	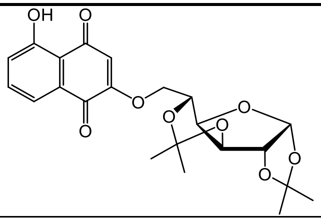	4.51	4.12	0.91
**7**	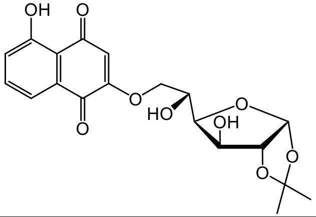	6.10	7.29	1.20
**8**	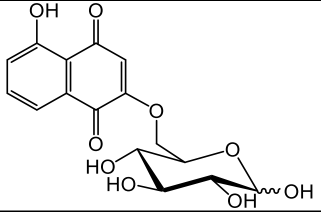	36.92	73.69	2.00
**9**	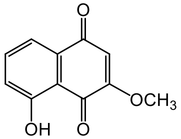	2.85	4.05	1.42
**10**	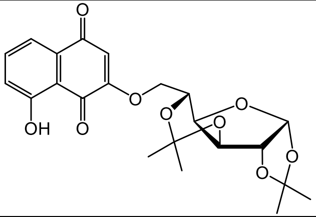	4.16	6.37	1.53
**11**	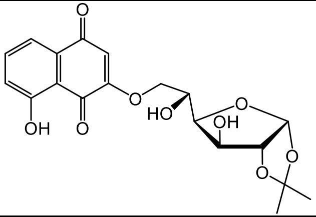	2.07	4.962	2.40

**12**	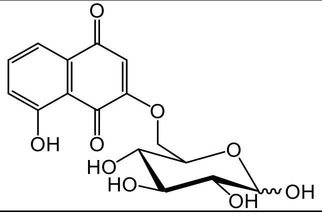	27.34	102.76	3.76
**13**	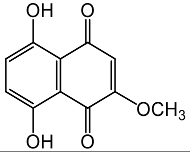	5.38	9.841	1.83
**14**	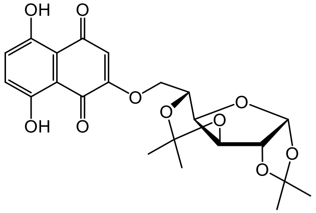	17.7	18.57	1.05
**15**	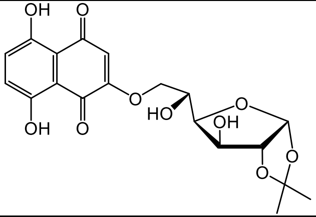	5.46	5.65	1.03
**16**	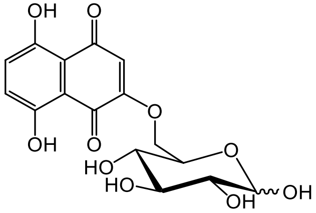	>100	>100	#
**17**	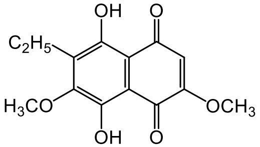	21.8	12.5	0.57

**18**	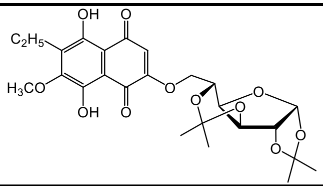	13.6	9.56	0.7
**19**	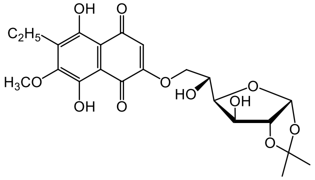	27.93	41.7	1.49
**20**	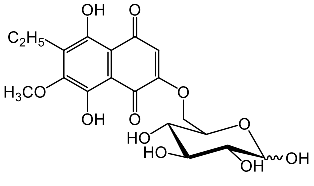	99	27.91	0.28
**21**	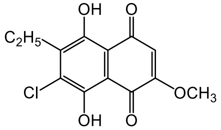	31.57	27.37	0.87
**22**	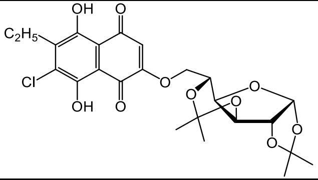	4.24	2.98	0.7
**23**	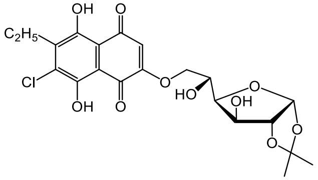	7.35	5.21	0.71

**24**	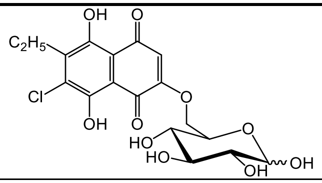	>100	>100	#
**25**	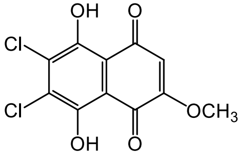	2.30	1.22	0.53
**26**	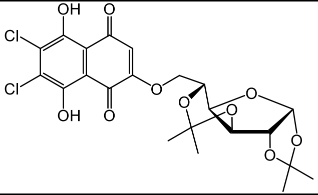	2.75	2.07	0.75
**27**	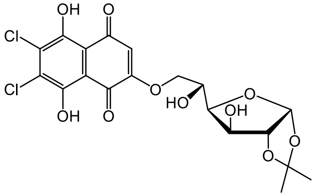	3.47	4.47	1.29
**28**	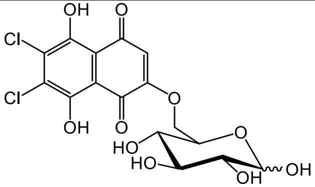	>100	>100	#
**Anisomycin**	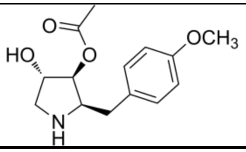	1.49	0.70	0.47

Table S5. The regulated proteins discovered by proteome analysis. Detailed information of Table S5 can be found at Supplementary Table S5.

Original Western Blotting Files

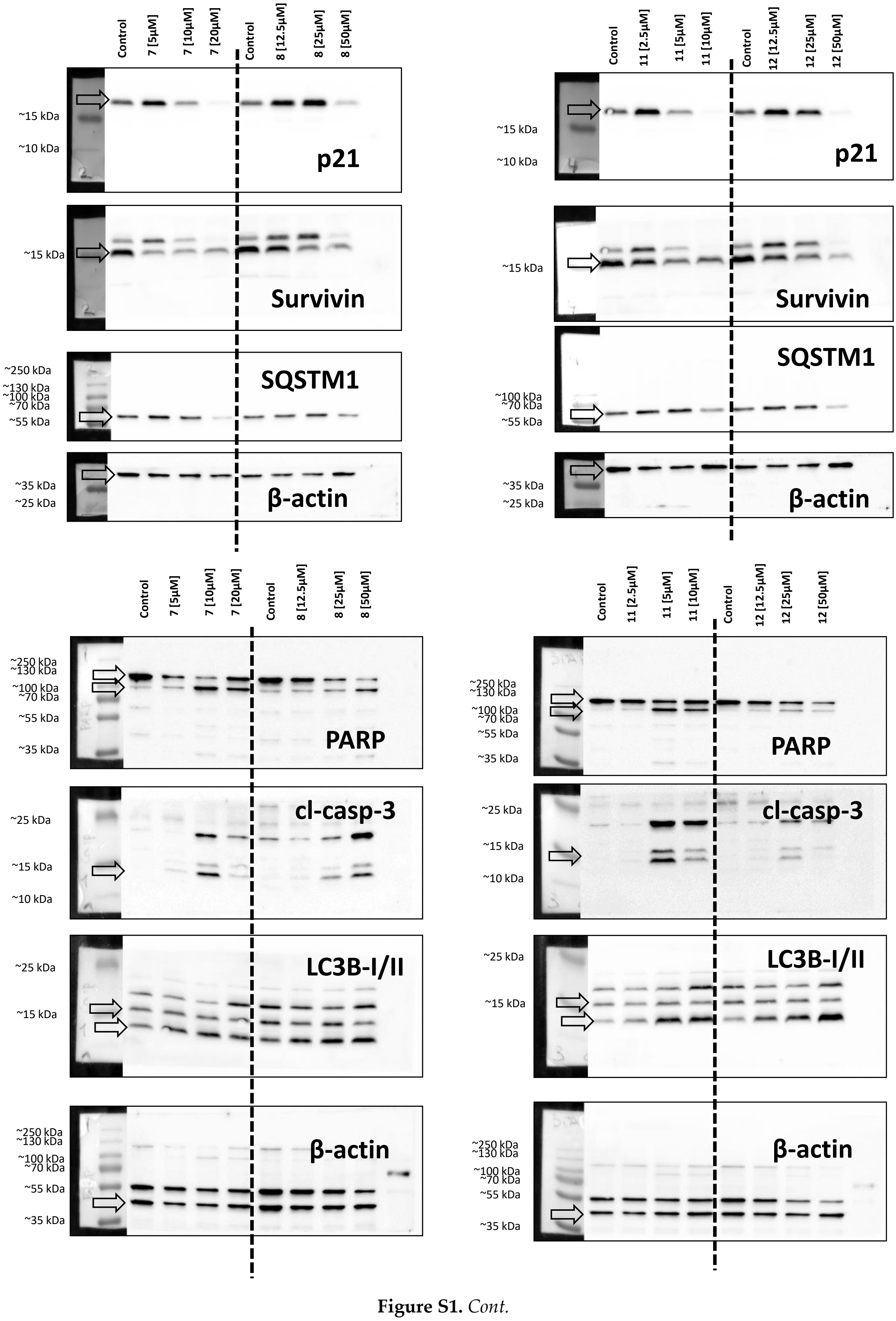

Figure S1. *Cont.*

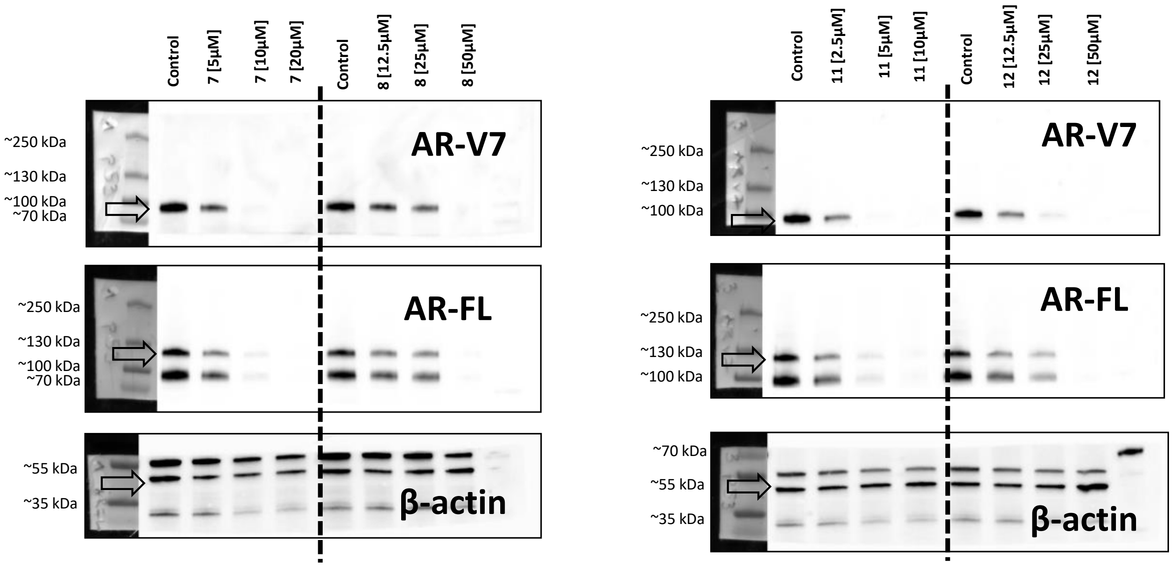

Figure S1. Original files for Figure 3F, G (Western blotting).

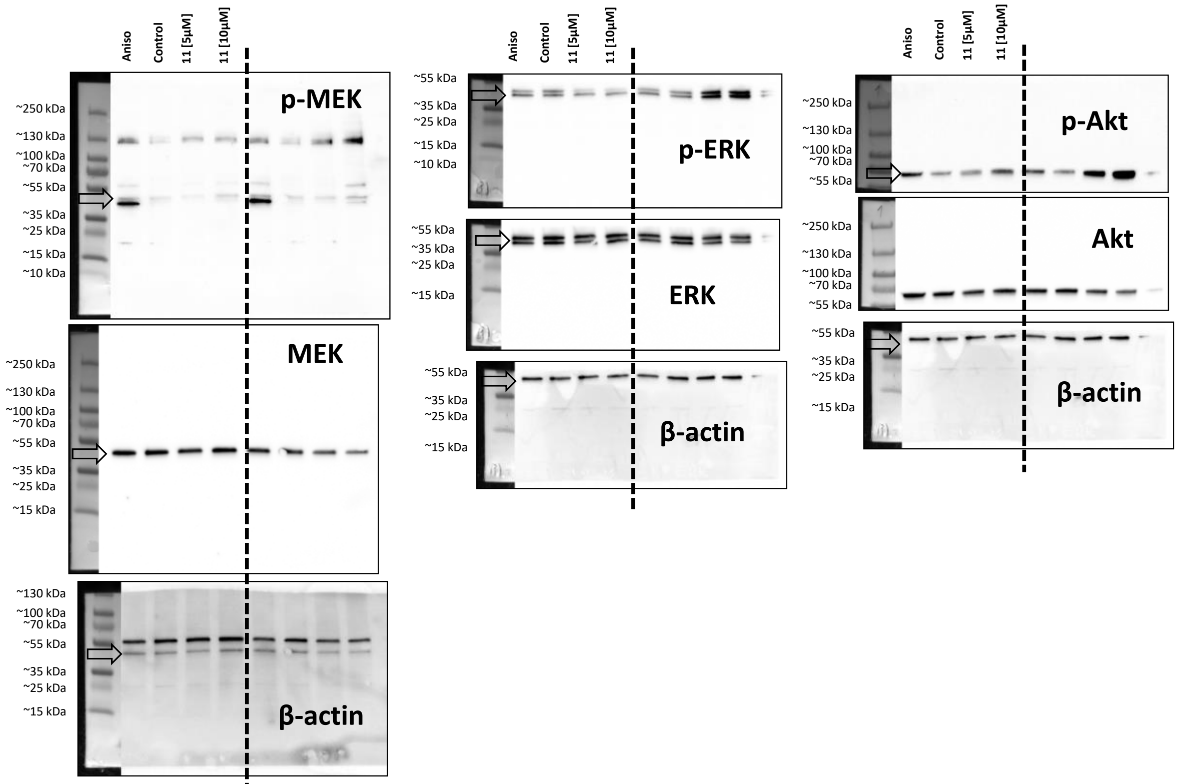

Figure S2. *Cont.*

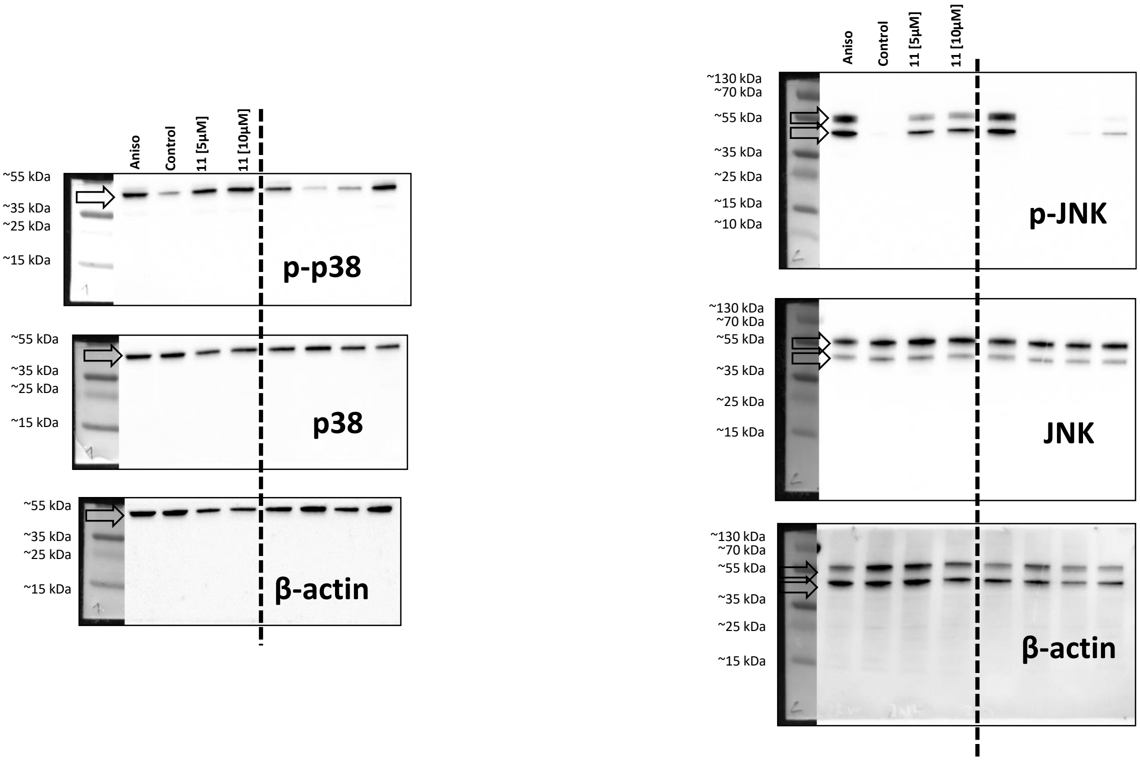

Figure S2. Original files for Figure 4E (Western blotting).

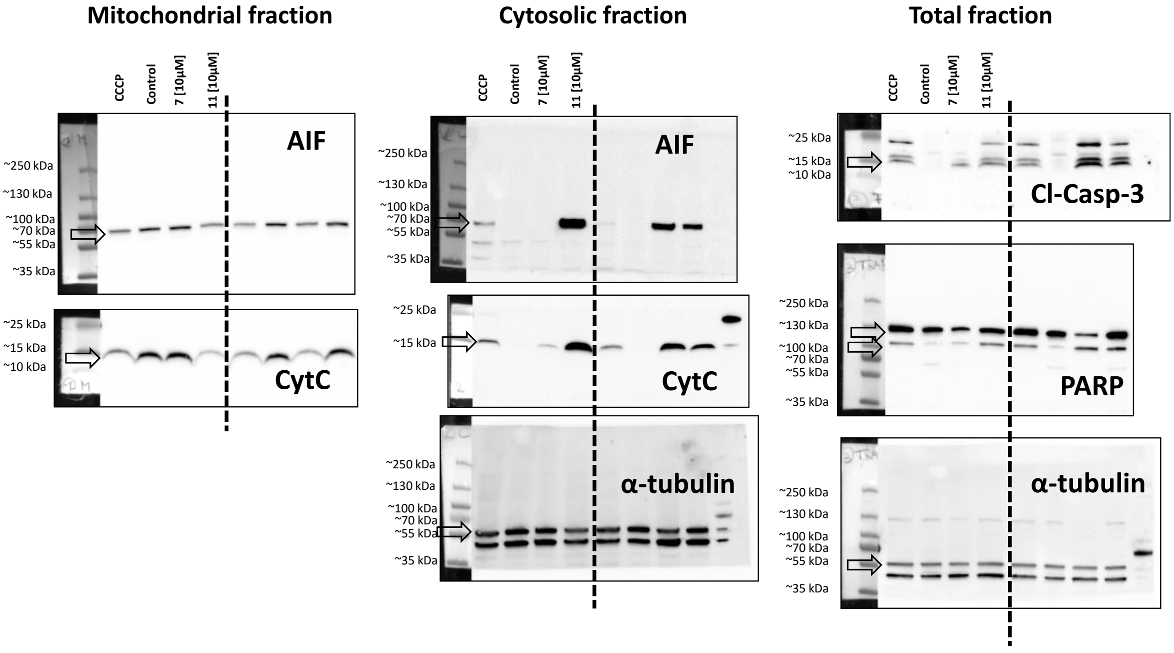

Figure S3. Original files for Figure 5F (Western blotting).

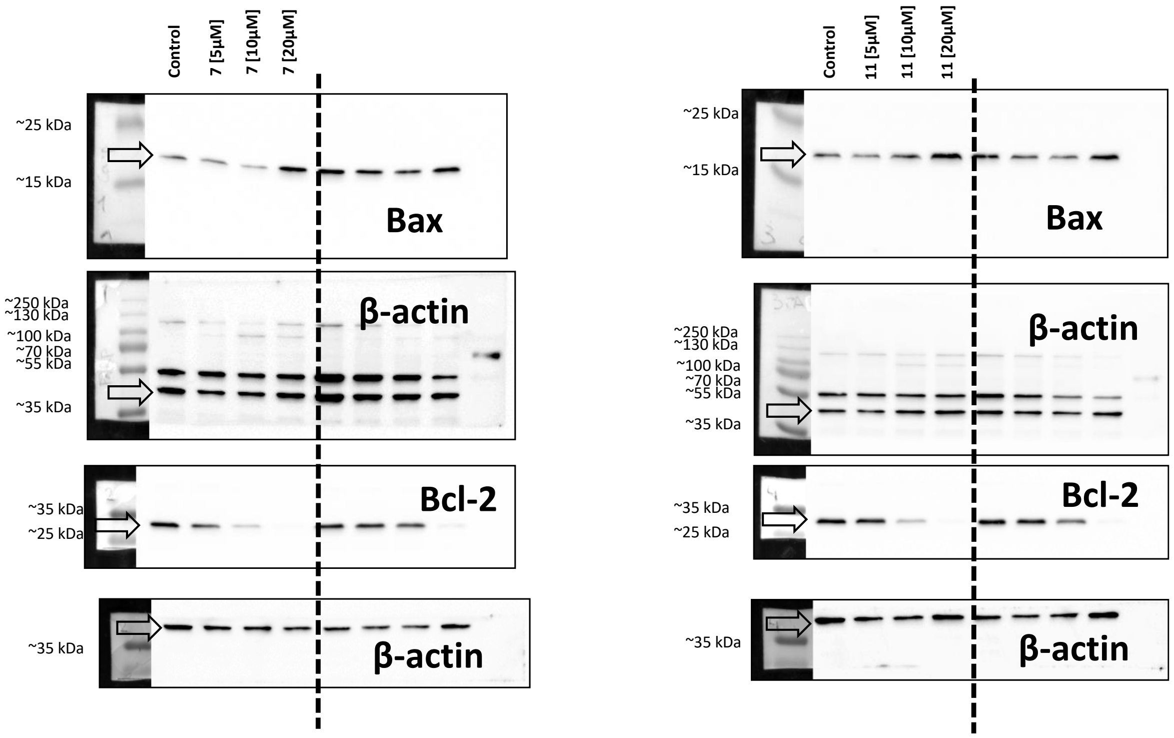

Figure S4. Original files for Figure 5G (Western blotting).

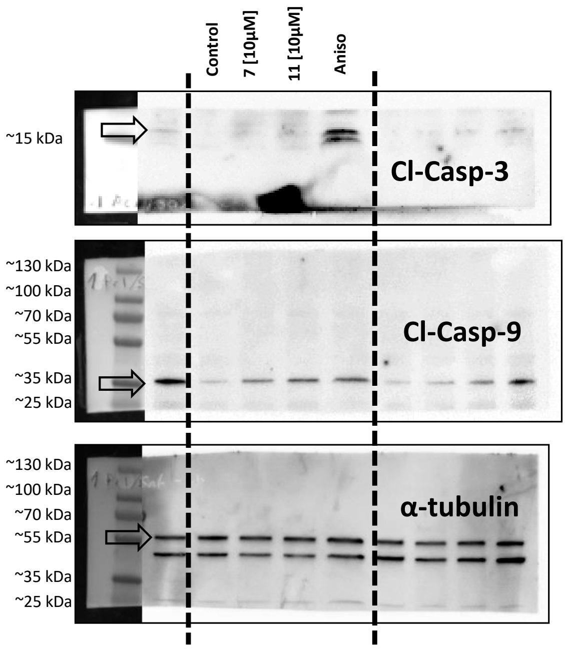

Figure S5. Original files for Figure 5H (Western blotting).

© 2019 by the authors. Licensee MDPI, Basel, Switzerland. This article is an open access article distributed under the terms and conditions of the Creative Commons Attribution (CC BY) license (http://creativecommons.org/licenses/by/4.0/).
